# A Comprehensive Review of Incorporating Steel Fibers of Waste Tires in Cement Composites and Its Applications

**DOI:** 10.3390/ma15217420

**Published:** 2022-10-22

**Authors:** Asad Zia, Zhang Pu, Ivan Holly, Tariq Umar, Muhammad Atiq Ur Rehman Tariq, Muhammad Sufian

**Affiliations:** 1 School of Water Conservancy and Civil Engineering, Zhengzhou University, Zhengzhou 450001, China; 2 Department of Concrete Structures and Bridges, Slovak University of Technology, 811 07 Bratislava, Slovakia; 3 Department of Architecture and the Built Environment, Faculty of Environment and Technology, University of the West of England, Bristol BS16 1QY, UK; 4 College of Engineering and Science, Victoria University, P.O. Box 14428, Melbourne 8001, Australia; 5 Institute for Sustainable Industries & Liveable Cities, Victoria University, P.O. Box 14428, Melbourne 8001, Australia; 6 School of Civil Engineering, Southeast University, Nanjing 210096, China

**Keywords:** sustainable environment, raw steel fibers recovered from waste tires, concrete, mortars, fiber reinforced concrete, mechanical strength

## Abstract

Accumulating vast amounts of pollutants drives modern civilization toward sustainable development. Construction waste is one of the prominent issues impeding progress toward net-zero. Pollutants must be utilized in constructing civil engineering structures for a green ecosystem. On the other hand, large-scale production of industrial steel fibers (ISFs) causes significant damage to the goal of a sustainable environment. Recycled steel fibers (RSFs) from waste tires have been suggested to replace ISFs. This research critically examines RSF’s application in the mechanical properties’ improvement of concrete and mortar. A statistical analysis of dimensional parameters of RSFs, their properties, and their use in manufacturing various cement-based composites are given. Furthermore, comparative assessments are carried out among the improvements in compressive, split tensile, and flexural strengths of plain and RSF-incorporated concrete and mortar. In addition, the optimum contents of RSF for each strength property are also discussed. The influence of RSFs parameters on various strength properties of concrete and mortars is discussed. The possible applications of RSF for various civil engineering structures are reviewed. The limitations and errors noticed in previous review papers are also outlined. It is found that the maximum enhancement in compressive strength (CS), split tensile strength (STS), and flexure strength (FS) are 78%, 149%, and 157%, respectively, with the addition of RSF into concrete. RSF increased cement mortars’ CS, STS, and FS by 46%, 50.6%, and 69%, respectively. The current study encourages the building sector to use RSFs for sustainable concrete.

## 1. Introduction

### 1.1. Background

#### 1.1.1. Tires and Global Pollution

Great attention on the global increase in tires is required, especially in the European Union [1,2]. More than one billion tires are used for replacement every year globally, with more than half being abandoned and waiting to be disposed of [3]. According to the European Tire and Rubber Manufacturers Association (ETRMA2021), its members manufactured 4.24 million tons of tires in 2021, representing 70% of the global tire industry’s turnover [4]. The European Union’s Landfill Directive [5], which went into force in July 2006, requires all end-of-life tires (ELTs) to be recycled or reused. Following the implementation of this regulation, ETRMA estimates that 91% of end-of-life tires (ELTs) were collected and treated for material recycling and energy recovery in 2018 [6]. About 40 million worn tires are processed annually in the United Kingdom alone. Material recovery, which uses secondary materials from ELTs in construction, automotive, and civil engineering applications, was used to treat around 2 million tons (61.75 percent of total ELTs handled). Apart from this, in 2009, China’s tire production by rubber consumption accounted for around 70% of the country’s total rubber resource consumption, resulting in 233 million waste tires weighing approximately 8.6 million tons, equivalent to approximately 3 million tons of rubber resources [7]. According to Pilakoutas et al. [8], over a billion discarded tires are produced worldwide. The accumulation of these tires is a significant difficulty because tire component materials are exceedingly complex, making natural degradation impossible [9]. This necessitates the careful management of this massive quantity of trash. Waste tires can be managed using various methods, including material recovery, energy recovery, retreating, export, and landfill disposal [8]. According to ETRMA 2018 [10], approximately 72% of waste tires in Slovakia were recycled, with zero percent of waste tires used in civil engineering, public works, or backfilling. As a result, investigating the potential application of waste tires or their ingredients in the construction industry to increase waste tire recycling in Slovakia and other countries is critical. Rubber content such as carbon black, steel insert, oil, and vulcanizing agents, such as inserts, synthetic yarns, and textiles are among the components of a tire, with percentages of 46 and 48 percent, 25–28 percent, 10–12 percent, and 3–6 percent, respectively [11]. The amount of steel fiber removed from waste tires varies according to the tire type. Steel is used in tires for up to 15% of lightweight vehicles, and up to 25% in trucks [8]. Liew and Akbar [12] reported that beneficial products from recycling tires consist of rubber, textile fibers, and steel fibers. Rubber makes up the majority of the tire, accounting for nearly 47–48 percent of the total weight, followed by black carbon (22 percent), steel cords (15–17 percent), textile fabric (5 percent), additives (8 percent), and zinc oxide and sulfur (1 percent each) [13,14].

#### 1.1.2. Raw Steel Fiber from Waste Tires and Concrete Composites

Concrete is extensively used in building materials because of its high compressive strength, durability, and environmental compatibility. Concrete is used in various architectural projects, including foundations, walls, bridges, roadways, dams, and reservoirs [15]. However, its service life may be severely shortened in demanding conditions. Although concrete structures usually are designed and built to last at least 50 years, sulfuric acid attack can cause them to deteriorate in just a few years. Repair and, in some cases, complete replacement of damaged structures are necessary when corrosion rates rise, which can be very costly and entail many social issues. Many research efforts have been made to improve concrete qualities for greater applicability due to these varied uses [16,17,18]. Industrial steel fiber reinforced concrete (ISFRC) has been beneficial in various applications throughout the last three decades, including tunnel linings, hydraulic structures, slabs, bridge decks, foundations, refractory concrete fiber shotcrete, and precast parts [8]. Adding 1% industrial steel fiber (ISF) to concrete automatically doubles the material cost [19]. As a result, steel fiber collected from discarded tires has become a viable option for use as reinforcement for cement composites. This advocates an environmentally beneficial method of dealing with some of the issues linked with the generation of waste tires. It also functions as a tool for improving building sector sustainability [20].

Pawelska-Mazur and Kaszynska [21] compared the energy consumption and CO_2_ emissions of the concrete mixture with raw steel fibers recovered from waste tires (RSF) to a concrete recipe with industrial steel fibers (ISF). It was concluded that the concrete recipe with RSF uses 31.3 percent less energy and emits 30.8 percent less CO_2_. However, due to intricate and energy-intensive manufacturing procedures, industrially manufactured steel fibers are the second component (after cement) that substantially impacts the natural environment. Therefore, energy consumption and greenhouse gas emissions could be reduced by substituting waste fibers obtained from the recovery of rubber from used tires for industrially manufactured steel fibers. 

Qin and Kaewunruen [2] demonstrated (refere to Table 7 in Qin and Kaewunruen [2]’s paper) that the RSF is far cheaper than ISF. The average cost of ISF is over five times that of RSF. Furthermore, replacing the ISF with RSF can reduce the cost from 15.89 USD/m^3^ to 40 USD/m^3^. According to the findings, the contribution of employing RSF substitutes for ISF in terms of economy and environment is significant. Furthermore, RSF’s social role cannot be overlooked. Waste tires pose a significant environmental problem [3]. Human health and water resources are irreversibly harmed by the combustion or burying of waste tires [22]. As an environmentally acceptable resource in civil construction, scrap tires improve building cleanliness and extend the structure’s service life. Even though RSF’s performance is neither higher than nor inferior to ISF concrete, it can still be a good substitute for ISF in economics, the environment, carbon emissions, and social development. According to concrete studies, replacing part of the ISF with some RSF is a good solution for waste tire disposal. Using RSF can produce similar advantages to using ISF on concrete [23]. Furthermore, 1.5 percent of RSF adds roughly 35 percent to construction budgets, and 1.5 percent of ISF contributes more than 50 percent [24]. In addition, the contribution of fiber to carbon emissions in concrete ranged from 15% (1.5 percent RSF) to 40% (1.5 percent ISF) [24]. According to a prior study, the RSF performs similarly to the ISF in enhancing concrete splitting and flexural strengths [25,26,27,28,29]. 

#### 1.1.3. Shortcomings in the Available Review Papers Related to RSF

Various mistakes were noticed in the previous review papers related to the possible use of raw steel fiber recovered from waste tires (RSF) in concrete. In some review papers, the steel fibers from industrial waste and lath machines are combinedly presented with that of raw steel fiber recovered from waste tires. For example, Liew and Akbar [12] reported that [30] the compressive strength of concrete increased by 59% when using RSF. But the authors used industrial waste fibers instead of RSF [30] in their research reported in the original paper. Similarly, Ahmed et al. [31] discussed the recycled steel fibers produced as a byproduct from Lathe or Turnery machines as RSFs, and conclusions were drawn related to optimizing the content of the RSF for better compressive strengths. 

In some papers, the RSF content of mortars is referenced for concluding the optimized content of RSF for concrete. For example, Liew and Akbar [12] and Ahmed et al. [31] reported that the research carried out by [32] showed that 45 kg/m^3^ of RSF increased the compressive strength of the concrete. However, research by the authors [32] was related to the impact of RSF on cement mortars, not concrete. Similarly, the research of [33] was related to incorporating RSF in mortars, and [12] reported it as concrete.

Some review papers dealt with only limited studies of the considered spectrum for a conclusion, such as Awolusi et al. [34], who based the review paper on the results of only four papers when comparing the mechanical properties. The authors [34] presented the results of only four papers (Nasir [19], Ndayambaje [35], Mastali and Dalvand [36], Syaidathul and Izni [37]) for analyzing the effect of RSF on the mechanical strengths (compressive, split tensile, and flexure strengths). Out of four, only two research studies (Nasir; Syaidathul and Izni) studied the effect of RSF on normal concrete. The other two studies (Ndayambaje; Mastali and Dalvand) considered the effect of RSF on rubberized concrete. The authors [34] did not provide the percent increase or decrease in the corresponding strengths due to the usage of RSF. They only mention the maximum strength obtained by the research in their studies, rather than the amount of improvement achieved by incorporating the RSF in concrete. 

The Balea et al. [38] review paper also comprises some limitations. The authors explored the outcome of all recycled fibers for the sustainability of hybrid fiber-reinforced concrete and gave a general and short review of the possible impact of RSF on the concrete. However, the in-depth discussion and improvement regarding use of the RSF’s specific content were not provided. The paper primarily focused on the impact of hybrid fibers (RSF in combination with other fibers). The review paper by Merli et al. [39] targeted the possible use of the different types of recycled fibers available and their properties, types, and generation process. But the impact of various dosages of RSF is not discussed, and a detailed analysis of the effect of RSF was missing. Ming et al. [40] also reviewed utilizing recycled waste fibers in cement composites, including RSF. The effect of RSF is discussed to a certain extent. However, a detailed comparative review related to the impact of RSF on the mechanical properties of the concrete was not reported. Instead, the general discussion was made by considering the previous studies on the application of RSF and other fibers for cement composites.

The given mistakes did not nullify the usefulness of the papers, but helped the readers read the papers while considering the mentioned mistakes in the view. Moreover, including the limitations mentioned above, the other problem with the previous review papers is that the effect of RSF is taken for both mortar and concrete. The influence of different doses of RSF independently on concrete and mortar has not been discussed and analyzed. In the current paper, the significance of RSF on the mechanical properties of concrete and zero coarse aggregate composite (mortar) are demonstrated independently. Secondly, the optimized contents of RSFs are discussed and analyzed separately for each composite for corresponding strength properties. Finally, the mistakes noted in the previous review papers related to different strength values are reported correctly.

## 2. Study Approach

This review primarily focuses on the mechanical properties of RSF-reinforced cement composites, i.e., concrete and mortar. Various search engines were used to find relevant literature. Papers were mainly collected using the bibliometric databases ScienceDirect, Google Scholar, Mendeley, and Web of Science, with three critical phrases used throughout searches: (1) recycled steel fiber; (2) waste tire steel fibers; and (3) waste steel fiber. 

As RSF-reinforced concrete is still a new topic, the review procedure used a cross-reference “snowballing” technique followed by Liew and Akbar [12]. Bibliographies of papers or citations to research articles were used in this strategy to broaden the range of relevant material in the research. The comprehensive search yielded more than 90 references on RSF-reinforced cement-based composites and their properties, which served as the foundation of the systematic literature. Next, the papers including incomplete information or not including plain concrete or mortar results as a reference were excluded. In addition, the papers including hybrid fibers (RSF in combination with fibers in the same mix) were also excluded. Papers from 2012 to 2022 were included.

Forty-three research studies were chosen for data analysis after seeking and gathering the most relevant publications. According to the compiled articles, the benefits of RSF in enhancing the mechanical characteristics of concrete and mortars may be accurately explained by analyzing data from various countries, authors, journals, and research institutes. In addition, its contribution to the performance of concrete and mortars was intended to be compared to that of plain concrete and plain mortar. Therefore, four steps were followed in the research order as given below:

- Collecting papers related to RSF-incorporated composites. Screening the most relevant papers using plain concrete or mortar as a reference.
- Arranging the material’s properties and strength properties of concrete and mortars separately.
- Determining the optimized content of RSF for each concrete and mortar and establishing the possible relationship between the strength properties of each of the composites with a dosage of RSF.
- Justifying the influence of RSF on a green and sustainable environment.

## 3. The Properties of RSF Fibers Used

A variety of RSF fibers have been used in various research studies. Overall, the diameters, lengths, tensile strengths, and aspect ratios of the RSFs used varied significantly.

Due to the extensive geometrical variation in recycled steel fibers, statistical analysis is required for frequency distribution and average fiber size for given numbers of representative fibers [2,41,42,43,44]. Raw steel fibers from waste tires can be straight or slightly twisted. Furthermore, different authors reported that the fibers could be categorized as macro or micro based on length [34]. Macrofibres range from 19 to 60 mm and are helpful for fracture bridging and structural support in hardened concrete [45]. Conversely, the length and diameter of microfibers are usually between 2–10 mm and 0.1–1 mm, respectively. 

The typical characteristics of the RSF used so far are reported in Table 1. The length of RSF fibers noted in previous papers extend from 7 mm to 101.16 mm. The average diameter varies from 0.15 mm to 1.4 mm. In comparison, the average tensile strength of the RSF varies from 781.3 MPa to 2570 MPa. The frequency of the various ranges of the diameter and lengths of the RSFs are also represented graphically.

**Figure 1:**
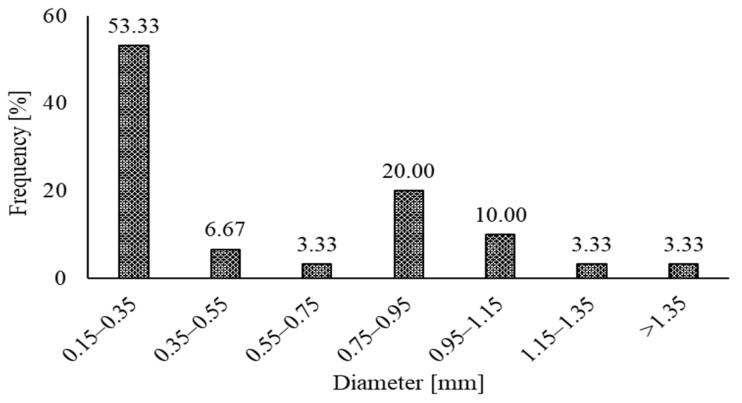
Frequency of RSFs for various ranges of diameters.

The frequency of the various ranges of diameter with a constant interval of 0.20 mm is shown in Figure 1. It can be noticed that the diameter of the number of fibers used in the previous papers mainly falls within the range of 0.15 mm to 0.35 mm. The fibers with a diameter of more than 0.95 mm were used in a small percentage of less than 17 percent.

**Figure 2:**
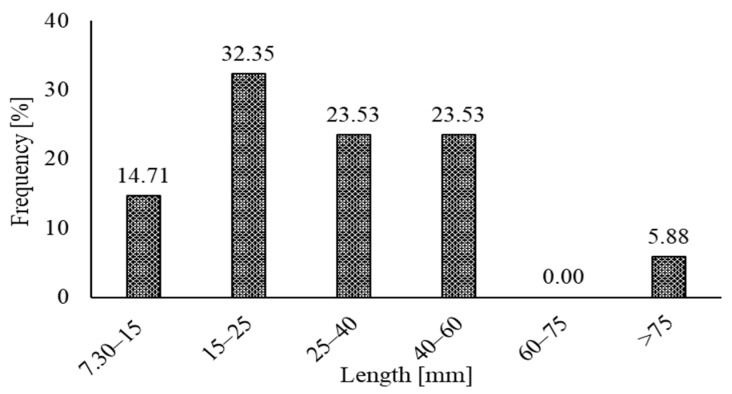
Frequency of RSFs for various ranges of lengths.

Similarly, the frequency of various ranges of the RSFs’ lengths with a constant interval of 15 mm other than the first interval, which is 8 mm, are shown in Figure 2. 

It is observed that a higher percentage (32.35%) of RSFs were used, whose length range was from 15 mm to 25 mm. The percentage of 23.57% of the lengths of the RSFs in the range of 40 mm to 60 mm is also quite considerable. However, tiny amounts (less than 5.88%) of fibers were used with an average diameter greater than 60 mm.

**Figure 3:**
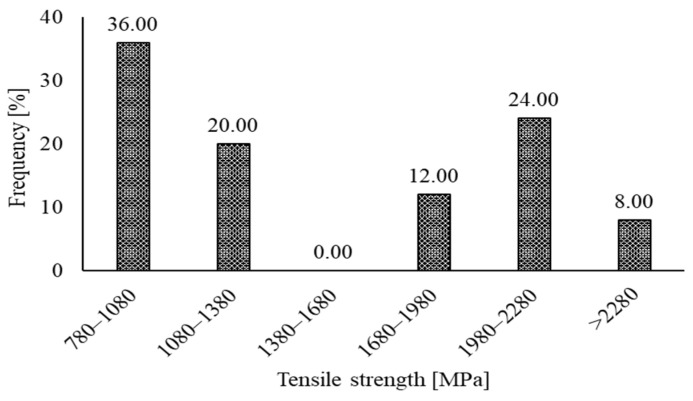
Frequencies of various tensile strengths of the RSFs.

The frequencies of the various ranges of the tensile strengths of the RSFs used in previous studies are demonstrated in Figure 3. RSFs with tensile strength ranging from 780 MPa to 1080 MPa were in the highest percentage of 36%. The second higher percentage of 24% was noted for the RSFs with tensile strength ranging from 1980 MPa to 2280 MPa. Finally, the minimum percentages of 0% and 8% are observed for RSFs used in previous studies, with a tensile strength range of 1380–1680 MPa and greater than 2280 MPa, respectively.

## 4. The Properties of RSF-Incorporated Concrete

The mechanical properties of the concrete incorporating raw steel fibers recovered from waste tires (RSFs) of previous research were demonstrated. Fundamentally, concrete is defined as a mixture of binder, fine aggregates, coarse aggregates, and water. Sometimes, additives or admixtures are added to obtain specific qualities. Hardened concrete’s three main mechanical properties are compressive strength, split tensile strength, and flexure strength. For percent comparison, the properties of the plain cement concrete of the concerned study were taken as a reference.

In some cases, it is challenging to compare the strength properties of different research studies due to differences in the scales used by authors for adding the fibers to the concrete. This becomes very difficult when the complete set of parameters of the fibers is not provided for converting their weight to volume or for interconversion among different scales of adding the fibers. To deal with this issue, the strength properties are compared separately for concrete containing RSF’s content by volume fraction of concrete, RSF’s content added by weight fraction of the concrete, and RSF’s content added in kg/m^3^. For this purpose, the strength properties of each category were compared individually, and the effect of variation of fiber was reported. In addition, an optimized dose for RSF was demonstrated for each of the RSF’s contents: fiber by volume or weight of concrete or in kg/m^3^. 

Most previous research has assumed that the density of RSF is the same as that of industrial steel fibers (ISFs), presuming that the RSF is free of impurities/tire particles or that impurities/tire particles have been separated from the RSF before mixing with concrete. However, recycled steel fibers from waste tires (RSF) were utilized in some studies without separating impurities/tire particles [25]. As a result, the average density of RSF with impurities/tire particles was determined to be less (3014 kg/m^3^) than the industrial steel fibers (7200 kg/m^3^) [25]. In addition, it found that the density of the RSF varies from that of the commonly used density of steel, 7850 kg/m^3^, for inter-conversion of steel weight and volume [63]. Therefore, it becomes hard to calculate the exact amount of RSF by volume fraction of concrete if the RSF fibers are added to concrete in kg/m^3^, and the density of the fibers is not given. Therefore, the properties of the effect of the addition of RSF in kg/m^3^ are discussed in a separate section.

### 4.1. Compressive Strength of RSF Concrete

#### 4.1.1. Compressive Strength of Concrete Containing RSF by its Volume Fraction

**Figure 4:**
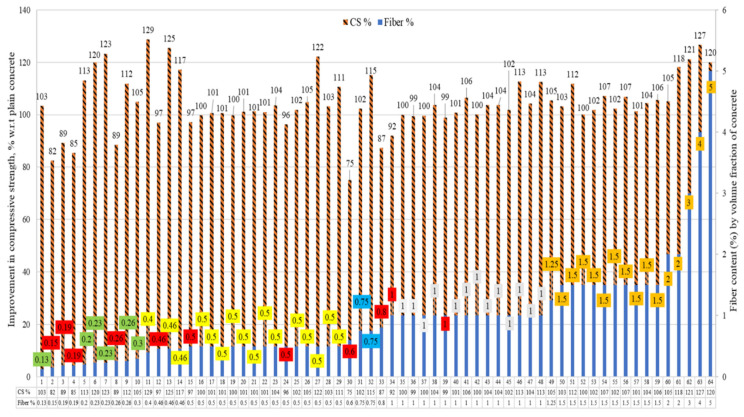
Percentage comparison of the compressive strengths of concrete containing RSF by volume fraction.

The compressive strengths (CS) of the concrete reinforced with raw steel fibers recovered from waste tires (RSF) were collected. The percent comparison of the CS is demonstrated in Figure 4. For percent comparison, the CS of the plain concrete of the concerned study was taken as a reference (equivalent to 100%). Additionally, the percent rise or decrease in the CS of RSF concrete is presented. 

The amount of fibers for specimens is given on the right axis of the graph, while an increase or decrease in CS to plain concrete is shown on the left y-axis of the graph. The read squares show the percentages of the fibers at which the CS was less than the PC. The rest of the different colors of the boxes represent a specific range of RSF doses used. The number of specimens is shown on the x-axis of the graph. Specimens 1 to 10 contain RSF from 0.13% to 0.30%. For specimen numbers 2, 3, 4, and 8, the CS decreased 18%, 11%, 15%, and 11%, respectively, from adding 0.15%, 0.19%, 0.19%, and 0.26% of RSF by volume fraction of concrete. The CS increased for specimens 1, 5, 6, 7, 9, and 10, by 3%, 13%, 20%, 23%, 12%, and 5% respectively. For RSF range from 0.46% to 0.50%, no significant decrease was reported except for specimens 12, 15, and 24, as shown in Figure 4.

Moreover, contradictory results were reported for specimen numbers 12, 13, and 14, incorporating the same amount of 0.46% RSF. The possible reason is that for specimens 13 and 14, the authors used a planetary concrete mixer; thus, good fiber dispersion and better mixing were obtained [48,66]. On the other hand, better mixing was not achieved for specimen 12 due to the traditional mixing method [41] used. 

At 0.50% of RSF, all researchers found no improvement or slight increase in CS of RSF specimens 15 to 29 except a slight decrease of 3% and 4% for specimens 15 and 24, respectively. Only a few studies (specimens 30, 31, 32, and 33) tested the CS of the specimen incorporating RSF from 0.60% to 0.80% by volume fraction of concrete. Increases of 2% and 15% were reported for the incorporation of 0.75% RSF in two studies. At the same time, decreases of 25% and 13% were reported for specimens 30 and 33, respectively, at 0.60% RSF and 0.80% RSF, respectively. A rise in CS of the RSF specimens was reported at 1% RSF for specimens 34 to 48, except for specimens 34 and 39. Specimen 34 and 39 showed a decline of 8% and 1% in CS at 1% RSF. No decline in CS was reported by any studies for specimens 49 to 64 for concrete incorporating RSF from 1.25% to 5% RSF by volume fraction.

The data given in Figure 4 is shown in tabular format for a specific range of RSF content, along with the diameters and lengths of the fibers. This can help conclude the influential factors for the increase and decrease in CS for the exact content of the RSF. The specimens incorporating RSF content of less than 1% are demonstrated in Table 2. The possible impact of the diameters and lengths can be described by comparing the results of the compressive strengths for the exact content of fibers in the context of RSF parameters. When advanced mixing techniques were not used, the concrete declined in compressive strength for short RSF (length was less than 31 mm) by more than 0.13%. A decrease in CS was reported for specimens 2, 3, 4, and 8, incorporating 0.13% to 0.26% RSF, by Aiello et al. [44]. The CS increased when RSF exceeded 0.19% for the exact dimension of the raw steel fibers, where special techniques were applied to control the fibers’ dispersion and homogeneity of the mix. Increases of 20%, 23%, and 12% in the CS of specimens six, seven, and nine for adding RSF of 0.23%, 0.23%, and 0.26%, respectively, could be correlated with using a planetary mixer and 0.17% more plasticizer.

Employing a planetary mixer for specimen preparation resulted in a considerable increase in the percentage of fibers supplied to the concrete mix and the dispersion of those fibers, thus significantly improving mix homogeneity. As a result, the blends were more workable for the same amount of water for specimens 6 and 7 [48,66]. On the other hand, Rossli and Ibrahim [37] reported an increase of 13% in the CS of the concrete at 0.20% RSF. The possible reason for this can be the good dispersion and use of long fibers (62 mm), which help in keeping the specimen intact and provide more resistance than the short fibers (26 mm) used by Aiello et al. [44]. Therefore, an increase in compressive strength can be expected even with less than 0.30% RSF if the homogeneity of the mix and dispersion of the fibers is controlled by using a particular procedure for mixing and preparation of the mix.

The RSF concrete specimens incorporating 0.40% to 0.50% of RSF were grouped. Significant decrease of 3%, 3%, and 4% were reported by Leone et al. [41], Centonze et al. (Centonze et al. 2012), and Siraj and Kedir [47], respectively, for 0.50%, 0.46%, and 0.50% of RSF. In contrast, other studies revealed an increase or negligible decrease in CS when adding 0.40% to 0.50% of RSF to concrete. For example, the highest increase of 29% was reported by Rossli and Ibrahim [37] for 0.40% RSF. The possible reason for obtaining a maximum increase with low RSF content of seems to be the considerable length (62 mm) of the sufficiently thick (0.80 mm) RSF used by the authors.

The second-highest increase in CS was noted by Skarzynski and Suchorzewski [54] for 0.50% RSF having moderate length and diameter of 26.17 mm and 0.25 mm, respectively. On the other hand, negligible improvement was reported by Vistos et al. [42] for 0.50% RSF having a small length and diameter of 12 mm and 0.27 mm, respectively. Therefore, it can be concluded that a considerable increase can be obtained in CS within the specified limit by increasing the dimensions of the fiber for the same amount of RSF. For RSF percentages of 0.60% and 0.80%, decreases of 25% and 13% in CS were reported for 62 mm length RSFs. In comparison, for short (7.30 mm) and medium-length (26.17 mm) RSFs, increases of 2% and 15% was observed for specimens 31 and 32, respectively, at 0.75% [52,58].

The specimens incorporating the RSF content equal to or more than 1% are demonstrated in Table 3. No decrease in CS was reported at 1% RSF, except 8%, 1%, and 1% decreases for specimens 30, 33, and 39, respectively. The possible reason for a decrease in the CS of the three specimens could be long fibers. Specimen 30, incorporating the longest fibers (62 mm), showed the highest decline in CS, while the other two showed a 1% decrease in CS for 60 mm fibers. The highest increase of 13% in CS at 1% RSF was reported for specimen 46, incorporating RSF had a length of 37 mm and diameter of 0.42 mm, and for specimen 48 [25] containing RSF with a length and diameter of 7.30 mm and 0.22 mm, respectively [55]. Hence, it can be suggested that the optimized content of the RSF can vary in RSF having different diameters for the same fiber length and vice versa.

For maximum CS within the same range, both the diameter and length of the fibers need to be considered. In the previous studies, an average increase of 2.53% was noted in CS for 1% RSF content having an average diameter of 38.18 mm and 0.79 mm. There was no decrease in CS for specimens incorporated with 1.25% to 2% RSF content. The highest increase of 27% was noted in specimen 63, which incorporated RSF, 40 mm long and 0.89 mm thick, as reported by Köroğlu [62].

The various lengths from 1.25% to 5% RSF were 7.30 mm, 20 mm, 30 mm, 40 mm, 45 mm, and 60 mm. The diameters of the RSF used were 0.22 mm and 0.245 mm for only specimens 49 and 61–64. While 0.89 mm thick RSFs were utilized in the rest of the specimens (50–60). The CS increased 9% at an average of 2.02% RSF, having an average length of 38 mm, and an average diameter of 1 mm. 

**Figure 5:**
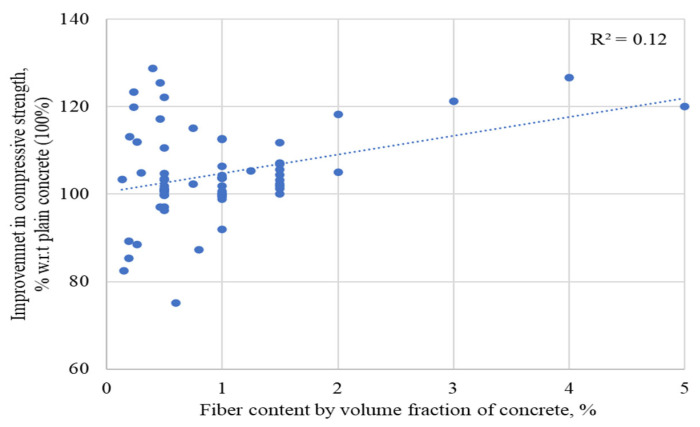
Percent comparison of RSF content with a percent increase in compressive strength of RSF concrete.

The percent improvement in compressive strengths of concrete incorporating RSF by its volume fraction is shown in Figure 5 compared to the percentage of RSFs used. It can be noted that most of the authors used various sizes of RSF in an amount less than 1.5% for evaluating the compressive behavior of RSF concrete. Few studies evaluated the effect of more than 2% of RSF having short-size fibers or RSF mix (containing various RSFs of different sizes). Nevertheless, the trend line shows the possibility of the CS being improved even at more than 1.5% of RSF. The available database’s regression coefficient (R2) is not very good (R^2^ = 0.12), but a reliable trend can be made possible in the near future with the comprehensive database of the compressive strength of concretes containing RSF by its volume fraction.

#### 4.1.2. Compressive Strength of Concrete Having RSF Content by Weight

**Figure 6:**
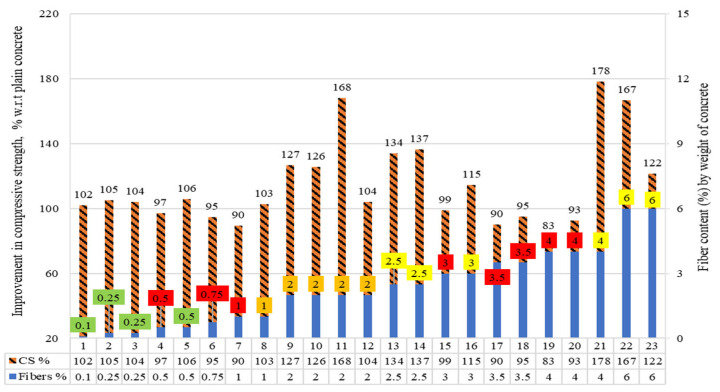
Percentage comparison of the compressive strengths of concrete containing RSF by its weight fraction.

The compressive strengths (CS) of the concrete, including raw steel fibers recovered from waste tires (RSF) by weight fraction, are presented in Figure 6. The CSs are compared, and a percent comparison is demonstrated. The CS of plain concrete from the associated research is used as a reference for percent comparison (equivalent to 100 percent). 

Also shown is the % increase or decrease in the CS of RSF concrete. The right axis of the graph shows the number of fibers in specimens, while the left y-axis shows the increase or decrease in CS to plain concrete. The x-axis of the graph represents the number of specimens. The percentages of fibers where the CS is less than the PC are displayed in the read squares. The remaining colored boxes each indicate a certain range of RSF dosages. RSF is present in 0.10 percent to 0.75 percent of specimens one through five. For 0.50 percent and 0.75 percent of RSF, the CS declined 3% and 5%, respectively. The CS increased by 2%, 5%, and 4%, respectively, for specimens one, two, three, and five. For 1% to 2% RSF, a significant increase in CS was reported for specimens eight to twelve, except for specimen seven. The authors reported a 5% decrease in CS at 1% RSF. By considering the specimen including RSF from 2.5% to 3.5%, a decrease of 1%, 10% and, 5%, in CS was reported for specimens 15, 17, and 18, respectively, for 3%, 3.5%, and 3.5% of RSF, correspondingly. An increase of 34%, 37%, and 15%, in CS was reported for specimens 13, 14, and 16, respectively, reinforced with 2.5%, 2.5%, and 3% RSF, compatibly. For specimens 19 and 20, 17% and 7% decreased at 4% RSF. In contrast, an improvement of 78%, 67%, and 33% was reported for specimens 21, 22, and 23, respectively, for concrete incorporating RSF of 4%, 6%, and 6%, respectively.

Table 4 demonstrates the results of the percent comparison and the properties of RSF used in the respective study. RSF concrete specimens with 0.20 percent to 0.75 percent RSF added by weight of concrete are grouped. Akhter et al. [61] and Shah et al. [60] noted increases of 2%, 5%, 4%, and 6% in CS for 0.10%, 0.25%, 0.25%, and 0.50% of RSF, respectively. 

A decline of 3% and 5% was reported by Shah et al. [60] for 0.50% and 0.75% RSF, respectively. Contradictory results were reported by Akhter et al. [61] and Shah et al. [60] for 0.50% RSF. The possible reason for improved CS could be better workability and uniformity achieved with reduced length (1 mm less) and diameters of the RSF utilized by Shah et al. [60]. A reduction in CS at 0.50% and 0.75% RSF can be associated with decreased workability due to excessive RSF within the same mix. The CS increased by 1.5% by incorporating 0.39% RSF in concrete by weight fraction. All authors reported an increase in CS by using 1% and 2% RSF except Shah et al. [60], who reported a 10% decline for specimen 7. 

Shah et al. [63] reported the same rhythm of decrease when RSF exceeded 0.25% of the concrete weight. At the same time, an increase in CS was reported for specimens 8 to 12, even for RSF having diameters of 100.16 mm. The maximum enhancement of 68 percent was noted in CS with 2% RSF, 29 mm long, and 0.20 mm thick [58]. Interestingly, Graeff et al. [59] and Gul et al. [46] reported an increase in CS for RSF with a minimum length of 13 mm and 7.62 mm, respectively. It can be deduced that an increase in CS can be achieved for the exact content of the RSF for varying dimensions of RSF. More RSF content can be used for small-size RSF compared to large-size RSF for achieving the same CS. For 2.5% RSF, the CS improved by 34% and 37% for specimens 13 and 14, while at 3% RSF, the CS decreased compared to 2.5% RSF concrete. A similar trend of decline in CS was reported for 3.5% RSF. However, it can be noticed that the short-length fibers could sustain a CS somewhat higher than the plain concrete at 2.5% RSF, while at 3% RSF, both types of RSF showed a decline in CS.

Therefore, maximum strength can be attained for 2% to 3% RSF by keeping the diameters of the fibers below 7.62 mm for traditional concrete mixers. A decline of 17% and 7% were reported in CS for specimens 19 and 20 when diameters of RSF were 100.16 mm and 7.62 mm, respectively. Short RSF showed less decline in CS. Interesting results are reported when 4% and 6% RSF had reduced diameters and lengths added to concrete. It was investigated that the highest increase of 78% in CS was achieved using 29 mm long RSF, which had a diameter of 0.20 mm. When the same RSF (29 mm long and 0.20 thick) was increased from 4% by weight of concrete to 6%, the increase in CS reduced from 78% to 67% [58]. An increase of 22% was noted by Graeff [59] when incorporating 6% of RSF with 13 mm length and 0.20 mm thickness. On average, no decline of CS was reported for 4% to 6% of RSF with a length of 13 mm to 29 mm and a diameter of 0.20 mm.

#### 4.1.3. Compressive Strength of Concrete Using RSF Content in Kg/m^3^

**Figure 7:**
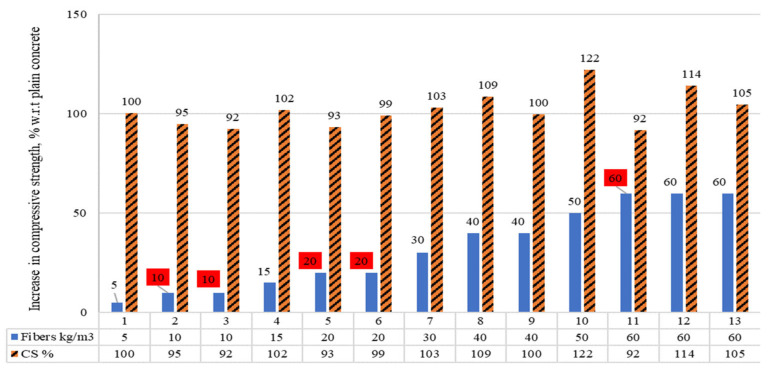
Percentage comparison of the compressive strengths of concrete containing RSF in kg/m^3^.

The compressive strengths (CS) of the concrete, containing raw steel fibers recovered from waste tires (RSF) in kg/m^3^, are demonstrated in Figure 7. The percentages of fibers where the CS was less than the PC are displayed in the read squares. A reduction in CS is noted for specimens 2, 3, 4, 5, 6, and 9 having RSF less than or equal to 20 kg/m^3^. However, a 3.2 percent increase was reported for specimen seven incorporating 30 kg/m^3^ RSF. Specimens reinforced with RSF of 30 kg/m^3^ to 60 kg/m^3^ showed a considerable rise in CS. Specimens 9 and 11 showed a decrease of about 1% and 8% in CS at 40 kg/m^3^ and 60 kg/m^3^, respectively. The data given in Figure 7 is presented in tabular format along with the length and diameters of the fibers used to assess the effect of the dimension of the RSF on compressive strength (CS) for the same amount of RSF. 

The given data are shown for a particular range of RSF content in Table 5, along with the diameter and length of the fibers. For 5 to 30 kg/m^3^ of RSF, no significant increase (more than three percent) in CS of concrete was reported except for a decline in CS. A decline of 2% is noted in the CS of concrete for average RSF content of 15.71 kg/m^3^ (5 to 20 kg/m^3^). This is noticed for 50 mm to 52 mm-long fiber, whose average diameter ranges from 0.30 mm to 1.40 mm. Therefore, it can be suggested that the RSF content below 20 kg/m^3^ cannot help to improve the CS of the concrete.

On the other hand, for the RSF ranging from 20 to 40 kg/m^3^, significant improvement was reported by the authors in CS, except for the 8.2% decrease reported by Sengul [49] for utilizing 60 kg/m^3^ of RSF. The possible reason for the decrease reported by [49] is the fibers’ large diameter (1.40 mm) and considerable length (50 mm). Therefore, the average length and diameters of the fibers used in the range from 20 kg/m^3^ to 60 kg/m^3^ are 38.75 mm and 0.81 mm, respectively. Hence, it can be concluded that 60 kg/m^3^ of RSF can be a suitable dose for improving the CS within the limited diameter and length of the fibers. However, in-depth analysis and research are still needed to optimize the suitable diameter and length of the fibers for the different ranges of RSF dosage.

### 4.2. Split Tensile Strength of RSF Concrete

#### 4.2.1. Split Tensile Strength of Concrete Using RSF Content by Its Volume Fraction

**Figure 8:**
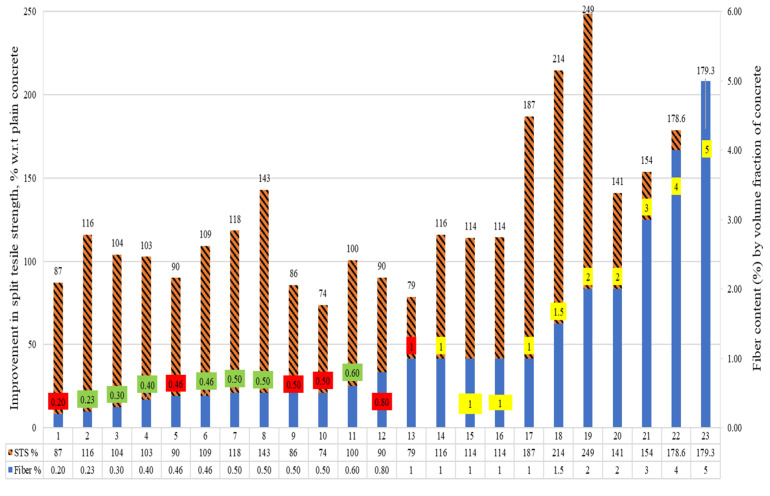
Percentage comparison of split tensile strength of concrete containing RSF by volume fraction.

The percent comparison of the split tensile strength (STS) of the concrete, including raw steel fibers recovered from waste tires (RSF) by its volume fraction, is presented in Figure 8. An inevitable decline was reported in STS when the content of RSF was lower than 1%. However, some specimens showed a substantial increase in STS even for RSF of less than 1%. The specimens 1, 5, 9, 10, and 11 incorporating RSF of 0.20%, 0.46%, 0.50%, 0.50%, and 0.80%, respectively, showed a decrease of 13%, 10%, 14%, 26%, and 10%, respectively, in the corresponding STS. On the other hand, specimens 2, 3, 4, 6, 7, and 8, containing 0.23%, 0.30%, 0.40%, 0.46%, 0.50%, and 0.50%, respectively, showed the substantial improvement of 16%, 4%, 3%, 9%, 18%, and 43%, in respective STS. No improvement in STS was reported for specimen 11 at 0.60% RSF. A decline of 10% in STS was noted only for specimen 13 at 1% of RSF. For more than 0.60% of RSF, no decline was reported. The authors noted a significant improvement in STS for adding 1%, 1.50%, 2%, 3%, 4%, and 5% of RSF, as reflected in Figure 8. In addition, the STS of the plain concrete of the concerned study is taken as a reference (equivalent to 100%) to assess the percent rise or decrease in the STS of RSF concrete.

A certain decline was reported in STS when the content of RSF was lower than 1%. However, some specimens showed a substantial increase in STS even for RSF of less than 1%. The specimens 1, 5, 9, 10, and 11 incorporating RSF of 0.20%, 0.46%, 0.50%, 0.50%, and 0.80%, respectively, showed a decrease of 13%, 10%, 14%, 26%, and 10%, respectively, in the corresponding STS. On other hand, specimens 2, 3, 4, 6, 7, and 8, containing 0.23%, 0.30%, 0.40%, 0.46%, 0.50%, and 0.50%, respectively, showed a substantial improvement of 16%, 4%, 3%, 9%, 18%, and 43%, in respective STS. No improvement in STS was reported for specimen 11 at 0.60% RSF. A decline of 10% in STS was noted only for specimen 13 at 1% of RSF. For more than 0.60% of RSF, no decline was reported. The authors noted a significant improvement in STS for adding 1%, 1.50%, 2%, 3%, 4%, and 5% of RSF, as reflected in Figure 8. 

The data regarding STS of RSF-incorporated concrete and the properties of RSF used in each study are in Table 6. This can help analyze the factors that can affect the split tensile strengths of concrete for the same percentage of RSF. For example, the effect of the fibers’ diameter and length can easily be observed on the STS of the concrete incorporating RSF ranging from 0.20% to 0.50%. Furthermore, it can be observed that a significant decrease, 13%, was noticed even for lengthy RSF at 0.20% [37] when traditional mixers were used.

In comparison, Aiello et al. [44] confirmed that an advanced vertical planetary concrete mixer substantially increases STS for 0.20% of RSF. In addition, a decline of 10% was reported in STS by utilizing 0.46% RSF having 14 mm length and 0.25 mm diameter [41]. In contrast, Aiello et al. [44] noted an increase of 16% in STS at the same amount of RSF (0.46%) by using lengthy (26 mm) fibers instead of 14 mm. The other possibility for an increase in the STS achieved by Aiello et al. [44] was using a planetary concrete mixer, which helped in the dispersion of fibers. Similarly, Dorr et al. [56] reported that the STS of RSF concrete declined by 14% for 0.50% of RSF when the best mixing method was not used. Overall, if reasonable care is taken during mixing and fibers are well dispersed, a relatively suitable STS increase can be achieved using 0.46% or more RSF. This is supported by the increase in STS reported by many researchers at 0.40% or more RSF by using a conventional concrete mixer [25,37,54]. At 1% RSF, a considerable increase of 16%, 14%, 14%, and 87% was noticed for specimens 14 to 17, respectively [25,37,53,62]). In contrast, Rossli and Ibrahim [37] stated that STS decreased by 10%, including 0.80% RSF. The results reported by Rossli and Ibrahim [37] contradict other research studies, which need to be cross-verified for possible justification. But Abdul Awal et al. [53] reported an increase in STS by increasing the dosage of the RSF to 2%.

An increase of more than 100% in STS was noted for specimens 18 and 19 using 1.5% and 2% of 30 mm long RSF. In comparison, a lower increase of 41% was reported in STS at 2% RSF [62]. Similarly, an increase of 54%, 78.6%, and 79.3%, was noted by incorporating 3%, 4%, and 5% to 45 mm-long RSFs in specimens 20, 21, and 22, respectively [62].

**Figure 9:**
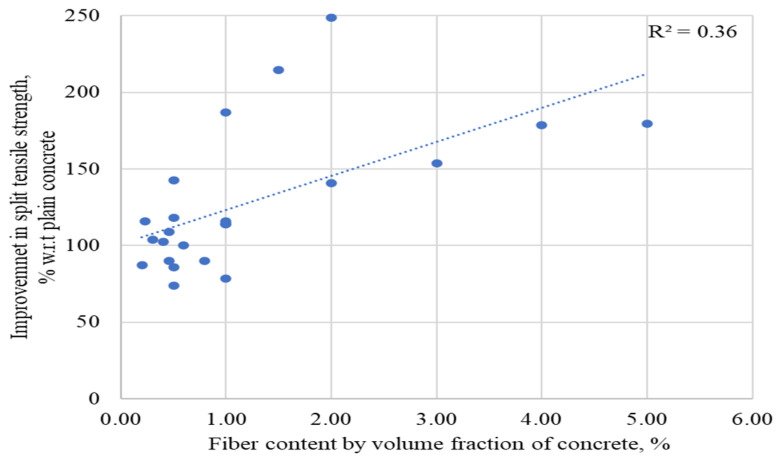
Percent comparison of RSF content with a percent increase in split tensile strength of RSF concrete.

Furthermore, exploring the relation between the content of RSFs used in previous studies and percentage improvements in split tensile strengths in corresponding research is also required. Therefore, the relation between percent improvement in STS of RSF concrete concerning plain concrete and the content of the RSFs is presented in Figure 9. It can be observed that most of the authors evaluated the split tensile behavior of RSF concrete using various sizes of RSF in amounts less than 1.5 percent. Few researchers looked at the impact of having more than 2% RSF with short fibers or an RSF mix (containing various RSFs of different sizes). The trend line illustrates that even at more than 1.5 percent RSF, the STS has the potential to improve.

The available database’s coefficient of regression (R^2^) is not very low (R^2^ = 0.36); however, an increased database of concrete STSs containing RSF by volume fraction can help obtain a clear image of the effect of more than 2% RSF. In addition, the various dimensional parameters of RSF must be considered to specify the trend of the increase in STS of the RSF concrete at a specific percentage of RSF.

#### 4.2.2. Split Tensile Strength of Concrete Using RSF Content by Weight

**Figure 10:**
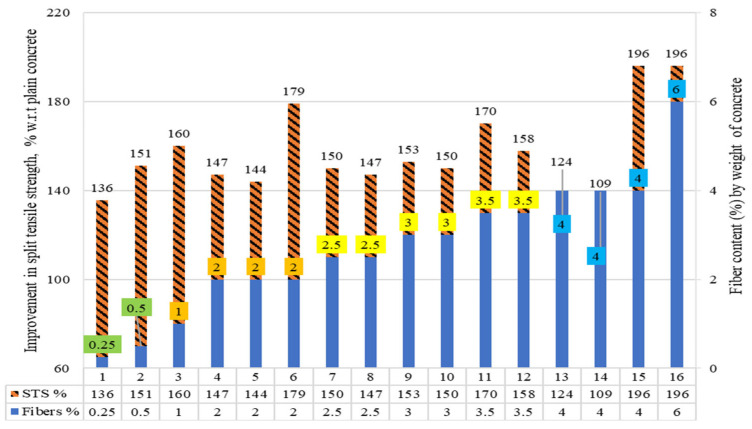
Percentage comparison of split tensile strength of concrete containing RSF by weight.

The split tensile strength (STS) of the concretes containing raw steel fibers recovered from waste tires (RSF) by their weight fraction is shown in Figure 10. The percentages of fibers where the STS was less than the PC are displayed in the read squares. The remaining colored boxes each indicate a certain range of RSF dosages.

The percent comparison among the results was revealed and discussed. The STS of the plain concrete of the concerned study was taken as a reference (equivalent to 100%) to assess the percent rise or decrease in the STS of RSF concrete. No decline was reported when including 0.25% to 6% of RSF in concrete by weight fraction. On the other hand, an increase of 9% to 96% was reported for RSF concrete compared to plain concrete. The STS of RSF-included concrete is summarized in Table 7, and the parameters of RSF employed in each study are also listed. This can assist in determining what factors influenced the split tensile strengths of concrete for the same RSF percentage. For example, maximum STS was reported for 1.1 mm thick RSF whose length was 30 mm when RSF varied from 0.25% to 1% by weight of concrete. 

Furthermore, when typical mixers were utilized, a considerable enhancement of 70.3 percent was seen in STS, even for long RSF (100 mm length) at 3.5 percent [46]. Younis [58] reported a maximum increase in STS by adding 2% of 29 mm long and 0.20 mm thick RSF. For 4% to 6% RSF, a decrease in STS was noted for specimens 13 and 14 compared to concrete, including same-size RSF in 3.5%. Still, the STS didn’t decline below the STS of plain concrete.

In contrast, a higher increase of 96% in split tensile strengths was noted for 4% and 6% RSF having small diameter and length compared to RSF of specimens 13 and 14. It showed that the diameter of the RSF can also have a detrimental effect on the concrete’s STS as the length of the RSF. Therefore, for optimizing RSF dose, the diameters of RSF need to be considered for the same length of RSF.

#### 4.2.3. Split Tensile Strength of Concrete Containing RSF Content in kg/m^3^

**Figure 11:**
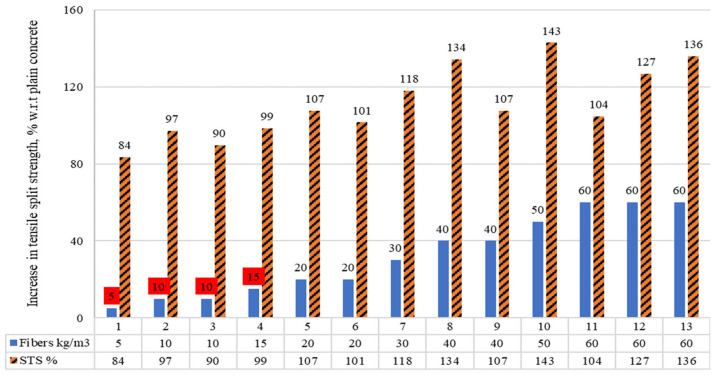
Percentage comparison of split tensile strengths of concrete containing RSF in kg/m^3^.

The split tensile strength (STS) of the concrete, including raw steel fibers recovered from waste tires (RSF) in kg/m^3^, is shown in Figure 11. Comparisons among the STSs are shown. The read squares show the percentages of fibers where the STS was less than the PC. For the percent rise or decrease in STS of RSF concrete, the STS of plain concrete in the concerned study was used as a reference (equal to 100 percent). It was noticed that significant improvement in the STS was not reported when using 20 kg/m^3^ or less than 20 kg/m^3^ of RSF except for specimen five, which showed a 7% rise in STS at 20 kg/m^3^ RSF. Most of the research studied demonstrated a noticeable increase in STS when incorporating more than 20 kg/m^3^ RSF. Specimens seven to thirteen showed an increment of 18%, 34%, 7%, 43%, 7.5%, 4%, 27%, and 36%, respectively, in their STS as compared to plain concrete. It showed the potential of the RSF to increase the split tensile strength of the concrete when the ratio of RSF was 20 kg/m^3^ or more. 

The data in Figure 11 are presented in Table 8, as well as the properties of the RSF used in the corresponding study. The table can help better understand the effect of RSF dimension on split tensile strength (STS) for the same range of RSF percentage. There was a decrease in STS of concrete with RSF 5 to 20 kg/m^3^, except for specimens five and six. The STS was improved when the content of the same RSF was increased for the same mix from 10 kg/m^3^ to 20 kg/m^3^ [49]. However, increasing the diameter of RSF for the same quantity decreased the STS of concrete as reported for specimen five (small diameter RSF) compared to specimen six (large diameter RSF). 

Overall, the average dose of 15.71 kg/m^3^ RSF with an average length and diameter of 50.86 mm and 0.59 mm reduced STS. However, for all reported diameters and lengths, the RSF ranging from 20 to 60 kg/m^3^, the authors noted a significant increase in STS. The highest increase of 43% in STS was reported by Pawelska-Mazur and Kaszynska [21] when RSF had 17.50 mm length and 0.25 mm diameter. It can be noted that the short fibers with a length less than 20 mm and a diameter less than 0.30 mm could be more beneficial in increasing the STS even at 60 kg/m^3^. For long fibers that were 50 mm long, the STS increased 34% at 40 kg/m^3^. When the content of the same-size fibers increases from 40 to 60 kg/m^3^, the STS enhancement lowers to 4% [49]. At the same 60 kg/m^3^ content of RSF, the 50 mm long and 1.40 mm thick RSF showed a lesser improvement of 4% in STS, and small-sized RSF (30–35 mm long and 1 mm thick) showed a higher enhancement of 27% and 36% for 30 mm long and 35 mm long RSFs, respectively. This showed that at the same proportion of RSF, an increase in STS can differ for different sizes of RSFs.

### 4.3. Flexure Strength of RSF Concrete

#### 4.3.1. Flexure Strength of Concrete Using RSF Content by Its Volume Fraction

**Figure 12:**
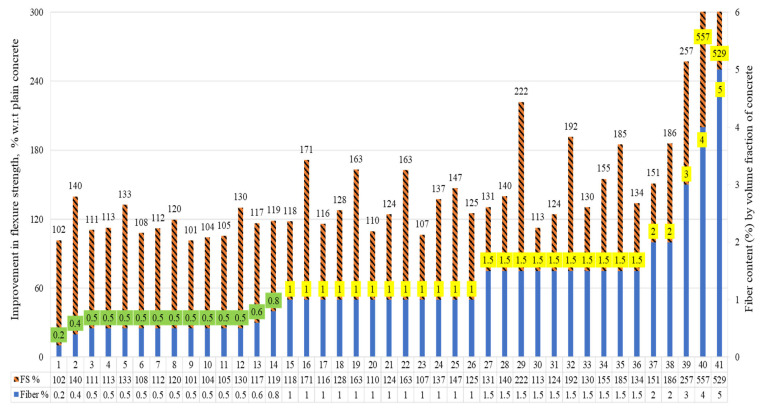
Percentage comparison of the flexure strength of concrete containing RSF by volume fraction.

The flexural strengths (FSs) of the concrete, in which the raw steel fibers recovered from waste tires (RSF) were added by its volume fraction, are demonstrated in Figure 12.

The percent increase and decrease are reported with reference to plain concrete. All specimens demonstrated a significant rise in FS with RSF of 0.20 percent to 5 percent. The contents of the RSFs used were 0.20%, 0.40%, 0.50%, 0.60%, 0.80%, 1%, 1.5%, 2%, 3%, 4%, and 5% by volume fraction of concrete. The enhancement in FS to plain concrete was 2% to 457% for RSF concrete.

The flexure strength of concrete specimens incorporating RSF by volume fraction and properties of RSF are provided in Table 9. The enhancement in FS increased from 2% to 40% when RSF enhanced from 0.2% to 0.4% and had a length and diameter of 62 mm and 0.80 mm, respectively [37], while the rise in FS was decreased when [37] increased the RSF content from 0.40% to 0.60% and 0.80%. A slight increase of 2% (117% to 119%) in FS was reported when RSF was increased from 0.60% to 0.80% for specimens 13 and 14, respectively. A considerable increase of 33% in FS was confirmed by [47] at 0.50% RSF for specimen five, for which the length and diameter of RSF were 60 mm and 0.89 mm, respectively. Siraj and Kedir [47] noted that within the range of 0.50% RSF, the longest (60 mm) fibers performed well in improving FS as compared to smaller (20 mm and 40 mm). 

An increment of 30% was also reported for specimen 12 at 0.50% RSF with 26.17 mm length and 0.25 mm diameter. This confirms that RSF can help improve the FS when added in a small dose of 0.4% by volume of concrete. While the long fibers can perform well, the maximum optimized length for better performance of the concrete in flexure still needs to be explored in depth. 

For specimens 15 to 41, the flexure strength increased by adding content of RSF from 1% to 1.5% for the same-size RSF [47]. Similarly, the increasing trend in flexure strength by increasing the RSF from 1% to 2% was also confirmed by Abdul Awal et al. [53]. For specimens 26, 36, and 37, an increase of 25%, 34%, and 51%, respectively, was observed for 1%, 1.5%, and 2%, respectively, of RSF.

Large increases of 157%, 457%, and 429% in FS were reported for specimens 38 to 41 for incorporation of 3%, 4%, and 5% of RSF, respectively [62]. All factors confirmed that a significant enhancement could be achieved for different dosages of RSF ranging from 0.4% to 2% by volume of concrete. The lengths of the RSF used in various studies were 20 mm, 26.17 mm, 30 mm, 40 mm, 60 mm, and 62 mm. The diameters of the RSF used were 0.25 mm, 0.80 mm, and 0.89 mm. The long-fiber (more than 62 mm) and thick-fiber (having a diameter of more than 0.89 mm) RSFs still need to be investigated to check their optimized content within 0.40% to 2%.

**Figure 13:**
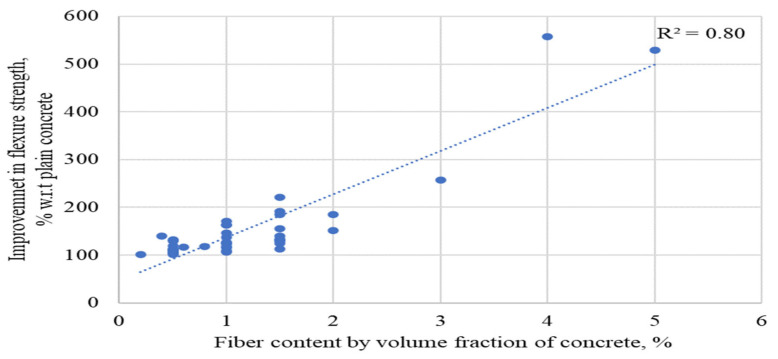
Percent comparison of RSF content with a percent increase in flexure strength of RSF concrete.

Investigating the relationship between the content of RSFs employed in prior experiments and % gains in flexure strengths in a subsequent study is necessary. Figure 13 shows the relationship between the percent changes in STS of RSF concrete compared to plain concrete and the RSF content by volume fraction of concrete. 

Most researchers examined the flexure behavior of RSF concrete using various sizes of RSF in amounts less than 1.5 percent, as can be seen. On the other hand, few studies have examined the effects of having more than 2% RSF with short fibers or an RSF mix (containing various RSFs of different sizes). Nevertheless, the trend line shows that the FS has the potential to improve at even more than 2 percent RSF. The graph’s coefficient of regression (R^2^) is good (R^2^ = 0.80); however, a more extensive database of split tensile testing results of concrete containing RSF by its volume fraction may assist in obtaining a clear picture of the effect for more than 2% RSF. Furthermore, the various RSF dimension aspects must be considered to specify the trend of the rise in STS of RSF concrete at a certain percentage of RSF.

#### 4.3.2. Flexure Strength of Concrete Using RSF Content by Weight Fraction

**Figure 14:**
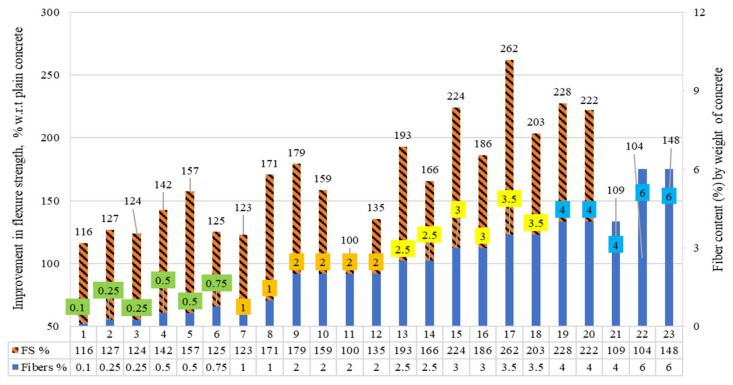
Percentage comparison of flexure strength of concrete containing RSF by weight.

Figure 14 shows the concrete’s flexural strengths (FSs), in which raw steel fibers recovered from waste tires (RSF) were added by the weight fraction of concrete. The percentage growth and reduction are presented in comparison to plain concrete.

By adding RSF 0.10 percent to 6%, all specimens showed a significant increase in FS except specimen 11. Only specimen 11 showed no improvement in FS for the inclusion of 2% of RSF by concrete weight.

Table 10 shows the flexure strength of concrete specimens possessing RSF by weight fraction and RSF characteristics. The FS of RSF concrete did not decline between the authors’ RSF ranges. When RSF climbed from 0.10 percent to 1 percent and had a length of 30 mm and 31 mm and diameter of 0.80 mm, enhancement in FS increased from 16% to 71%. 

When 30 mm long RSF content increased from 0.10 percent to 0.25 percent and 0.80 percent, improvement in FS compared to plain concrete increased from 16% to 27% [60]. While at the same 0.35% content of 31 mm long RSF, an increase of 24% was reported for specimen three [61]. Similarly, the improvement in FS was 42% and 57% for specimens four [60] and five [61] at 0.50% RSF. However, dissimilarity among the results was noted by researchers for 0.75% and 1% RSF, at 0.75% and 1% of 30 mm RSF. Compared to plain concrete, an enhancement in FS reduced from 42% (at 0.50% RSF) to 25% and 23% for specimens six and seven, respectively [60]. Hence, using 30 mm RSF, the FS started declining gradually to increase RSF from 0.50%. However, at the same 1% content of 31 mm RSF, an increase of 71% in FS was reported for specimen eight [61]. The possible reason can be a difference in the homogeneity of the mixture with each author, while the other possible reason could be the large 1.10 mm thickness of the RSF used in specimens six and seven [60]. However, both studies cannot be compared because the diameters of the fibers were not given for specimens six and seven [60].

For the RSF content of 2% to 3.5%, the maximum increase in FS was 162% compared to plain concrete at 3.5% RSF, which had 100.16 mm length and 0.94 mm thickness for specimen 17 [46]. At the percentage of RSF, an improvement of 101% was reported for 7.62 mm long and 0.94 mm thick RSF for specimen 18 [46]. This confirmed that decreasing the diameter of RSF for the same percentage of RSF could cause an inevitable decrease for the same concrete mixture. A similar decrease in FS for reducing the length of the fibers was supported by results reported for specimens 12 to 16 [46]. On the other hand, an incline of 35% was reported in FS, when 2% of small-size 13 mm long RSF had a diameter of 0.20 mm were incorporated in specimen 12 [59]. 

The contrasting results (no increase in FS) were reported for specimen 11 at 2% RSF (29 mm long and 0.20 mm thick) [58]. The possible reason could be low-strength concrete (FS = 4.60 MPa); thus, less cement paste was available to keep the mix homogeneous and uniform. For using 4% to 6% RSF, a significant increase in FS was reported for all specimens, as shown in the table. For the same-size RSF (100.16 mm long and 0.94 mm thick), the increase in FS reduced from 162% (specimen 17) to 128% (specimen 19) [46] by increasing the RSF content from 3.5% to 4%, respectively, while for the smaller-size RSF (7.62 mm and 0.94 mm diameter), the FS gradually improved from 103% to 122% for specimen 20 [46]. An increase of 9% was also reported for specimen 21 for low-strength concrete (5 MPa), incorporating 4% RSF, which was 29 mm in length and 0.20 mm in diameter [58]. However, the improvement in FS declined to 4% when the content of same-size RSF was increased to 6% for specimen 22 [58]. A similar trend of 35% and 48% increases in FS were noticed at 2% to 6% small-size RSF (13 mm length and 0.20 mm diameter), respectively, correspondingly for specimens 12 and 23 [59]. This showed that a significant enhancement in flexure strength could be achieved even at 6% RSF if rationally small-sized fibers are used, while the same strength can be achieved using less lengthy RSF in concrete. There is a dire need to evaluate the possible optimized content of RSF for the flexure strength of concrete by considering the specific size of the fibers. Still, more research studies are required to evaluate the effect of different contents of the same size of the RSF on flexure strength, particularly the addition of 5% or more of small-size RSF in concrete by weight proportion of concrete.

#### 4.3.3. Flexure Strength of Concrete Using RSF Content in kg/m^3^

**Figure 15:**
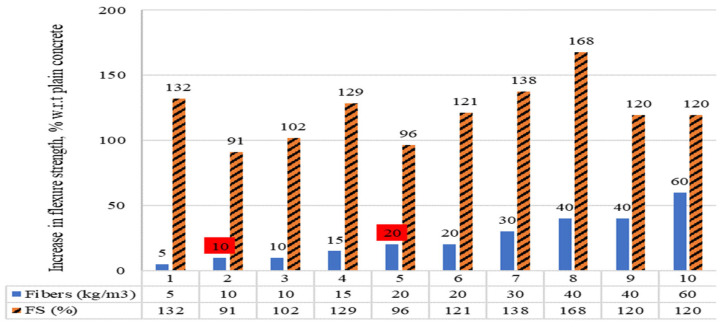
Percentage comparison of flexure strength of concrete containing RSF in kg/m^3^.

The percentage comparisons among the flexure strengths (FS) of concrete, in which raw steel fibers recovered from discarded tires (RSF) were added in kg/m^3^ are demonstrated in Figure 15. The percentage rise or decrease in FS of RSF concrete with respect to plain concrete is revealed. A 9% and 4% decrease in FS was noted for less than 20 kg/m^3^ RSF content for specimens two and five, respectively. For more than 20 kg/m^3^ of RSF, a decline in FS was not reported for any specimen. An increase of 32%, 29%, 2%, 21%, 38%, 68%, 20%, and 20% were reported for specimens 1, 4, 6, 7, 8, 9, and 10, respectively. 

The results presented in Figure 15 are illustrated in Table 11, along with the features of the RSF. An increase of 32% and 29% were reported at 5 kg/m^3^ and 15 kg/m^3^ of 52 mm long and 0.30 mm thick RSFs for specimens one and four, respectively. The 9% decrease in FS noted for same-size RSF at 10 kg/m^3^ was not well-justified by the authors. Similarly, a decline of 4% in FS noted at 20 kg/m^3^ RSF (50 mm long and 0.60 mm thick) for specimen five was not logically justified [49].

For the same-size RSF (50 mm long and 0.60 mm thick), an increase of 2% and 21% was noticed in FS at 10 kg/m^3^ and 20 kg/m^3^, respectively, for specimens three and six. It can be noted that at the lower percentage (5 kg/m^3^) for small-diameter (0.30 mm) RSF 32.1% increase in FS was noted, while the 4.5 times increase in RSF diameter (1.40 mm) showed little increase (21%) in FS at 20 kg/m^3^. This confirms that the diameter and length of raw steel fibers recovered from waste tires must be considered to optimize their flexure strength.

## 5. Properties of RSF-Incorporated Mortars

Like concrete, the hardened properties of the cement mortars incorporating raw steel fibers recovered from waste tires available in the previous papers were compared and discussed. Mortar is a mixture of binder, fine aggregates, and water. Sometimes, additives or admixtures are added to obtain unique characteristics. The effect of RSF on mortar is discussed separately because its composition and behavior differ from that of concrete. More cement paste is available for gripping raw steel fibers recovered from waste tires (RSF) than mortar concrete. Thus, the behavior and the effect of various contents of the RSF can be different for mortar than concrete. The three main mechanical properties are compressive strength, split tensile strength, and flexure strength. For percentage comparison, the properties of the plain cement mortars of the concerned study were taken as a reference.

### 5.1. Compressive Strength of RSF Mortars

**Figure 16:**
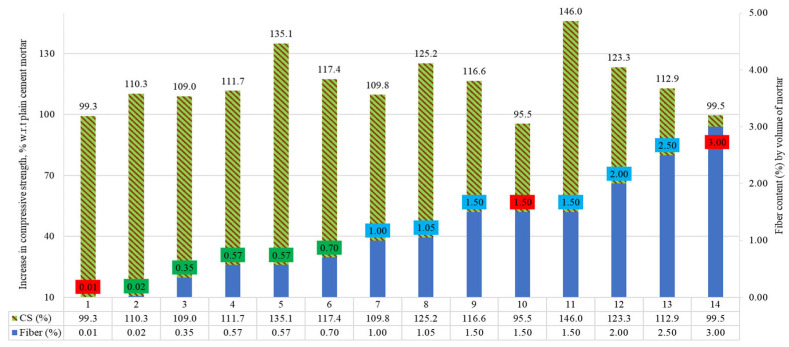
Percentage comparison of compressive strengths of mortars containing RSF by volume fraction.

The compressive strengths (CS) of the mortars incorporating raw steel fibers recovered from waste tires (RSF) by their volume fraction are discussed in this section. The percentage comparison of the compressive strengths is demonstrated in Figure 16. 

The read squares show the percentages of fibers where the CS was less than the PC. The other colored boxes represent other RSF dose ranges. For percentage comparison, the percentage increase or decrease in CS RSF mortars is presented with reference to the plain mortar of the corresponding study. A decline of 0.7%, 4.5%, and 0.5%, in CS was reported for three specimens (1, 10, and 14) incorporating 0.01%, 1.50%, and 3% of RSF, respectively. For other specimens, a significant increase in CS was reported. An increase of 10.3%, 9%, 11.7%, 35.1%, 17.4%, 9.8%, 25.2%, 16.6%, 46%, 23.3%, and 12.9% was noted in CS for specimens 2 to 9, and 11 to 13, respectively. Specimens 2 to 9 and 11 to 13, contained RSF of 0.02%, 0.35%, 0.57%, 0.57%, 0.70%, 2%, 2.05%, 1.50%, 1.50%, 2%, and 2.50%, respectively.

The compressive strength of mortar specimens with RSF by percent volume and RSF features are shown in Table 12. No significant change in compressive strength was reported for mortar specimen one at 0.01% for 14.9 mm long and 0.32 mm thick RSF. However, when RSF was increased in specimen two from 0.01% to 0.02% for the same-size (14.9 mm long and 0.32 mm thick) RSF, 10.3% was observed [66]. 

On the other hand, only a 3% increase in compressive strength was reported by using thirteen times more (0.13% by volume of concrete) of the lengthy RSF (26 mm long and 0.258 mm thick) in concrete with a high percentage (1.37%) of superplasticizer [44], while for a moderate superplasticizer (1% and 1.20%), no improvement in CS was reported for concrete when including less than 0.26% RSF [44]. This showed that a significant increase could be achieved in CS of the mortar with such a low amount of small-size RSF, which would not contribute to CS of the concrete in that amount. In contrast, a 9% increase was observed for specimen three, at 0.35% of RSF with 50 mm length and 0.15 mm diameter [52]. This highlights that decreasing the fibers’ diameter can adversely influence the compressive strength of the mortar for the same amount of RSF. Furthermore, although the length of RSF for specimen three was about two times more than RSF used in specimens two and one, an about two times reduction in diameter of the RSF caused a notable decrease in CS even at a higher dose of RSF. 

A maximum increase of 35.1% in CS was noted for RSF, ranging from 0.10% to 1% for specimen five, including 21 mm long and 0.20 mm thick RSF (Al-musawi et al. 2019). This validates the effect of RSF size on the CS for its same content. One percent to three percent of RSF, only for specimen 10, a decline of 4.5% was reported at 1.50% (Zamanzadeh et al. 2021). The possible reason for this decline can be associated with the large thickness (0.92 mm) of short-length (31 mm) RSF, which might be unable to contribute to the CS of the mix in such a low volume. In the same study, CS was almost equal to plain mortar when RSF was increased from 1.50% to 3% for specimen 14. On the other hand, significant enhancement in CS was observed for other specimens. A maximum of 46% improvement was noted for specimen 11, including 1.5% RSF, 50 mm length, and 0.15 mm thickness [24]. At the same 1.50% RSF (25 mm long and 0.26 mm thick), an improvement of 16.6% was reported for specimen nine (Dehghanpour and Yılmaz 2018). It can be deduced that at the same 1.50% RSF, an increase in CS was minor for small-size RSF. A similar increase in CS with large RSFs was confirmed by specimens seven (1% RSF) and eight (1.05%), which contained almost the same percentage of RSF. A decrease in CS was noted for increasing the same-size RSF from 2% to 2.5% for specimens 12 and 13 (Dehghanpour and Yılmaz 2018), while the opposite trend of increase in CS was stated for specimen 14 for very thick RSF at 3% content. In short, the current research related to applying various doses of RSF for CS improvement of cement mortars or zero self-compacting concrete (zero coarse aggregates) still needs a more in-depth experimental program to optimize various sizes of RSF. The current literature on RSF mortar is insufficient for concluding the optimized content of RSF for mortars.

### 5.2. Split Tensile Strength of RSF Mortars

**Figure 17:**
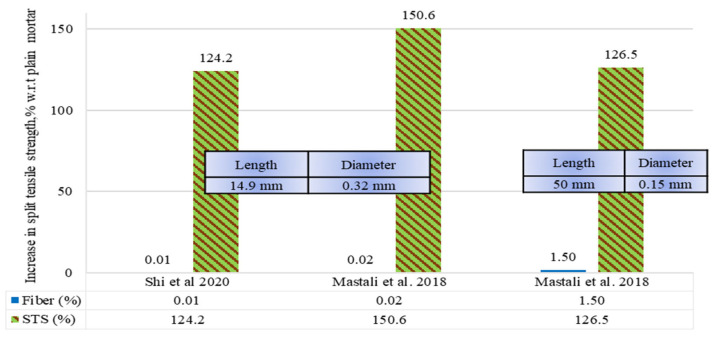
Percentage comparison of split tensile strengths of RSF mortars containing RSF by its volume fraction.

The percentage comparison of the split tensile strength (STS) of the mortars (zero coarse aggregate concrete) incorporating raw steel fibers recovered from waste tires (RSF) by volume fraction is demonstrated in Figure 17. In addition, the percentage increase or decrease for RSF mortars in STS is compared to plain cement mortar (100%). Few split tensile mortar samples have been tested for the possible influence of RSF inclusion on mortar. Significant improvements of 24.3%, 50.6%, and 26.5% were reported in STS for inclusion of 0.01%, 0.02%, and 1.50% RSF in mortars.

Opposite to concrete, the inclusion of small-sized RSF (14.9 mm long and 0.32 mm thick) also significantly improved STS. However, only a 16% increase in split tensile strength was reported when using twenty times the amount (0.2% by volume of concrete) of 11 mm longer RSF (26 mm long and 0.258 mm thick) in concrete [44]. The increase in STS was less (26.5%) when 1.50% of 50 mm long RSFs were incorporated in mortar. The effect of adding RSF by volume fraction of mortar still needs to be explored in more depth for the maximum possible split tensile strength.

### 5.3. Flexure Strength of RSF Mortars

**Figure 18:**
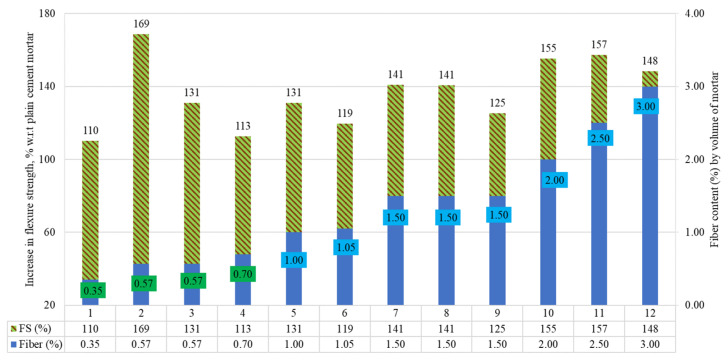
Percentage comparison of the flexure strength of RSF mortars encompassing RSF by volume fraction.

The percentage comparison of flexure strengths (FS) of the raw steel fibers recovered from waste tires (RSF) incorporated in mortar by volume fraction is demonstrated in Figure 18. The percentage inclines and declines are demonstrated in terms of plain cement mortars. An improvement of 10% to 69% in FS was reported for all specimens. No decline in FS was reported for the inclusion of various RSFs ranging from 0.35% to 3%. An enhancement of 10% to 69% was reported in FS for the inclusion of various amounts of RSFs. 

Table 13 displays mortar specimens’ flexure strength and RSF properties, including RSF by percent volume. Mortar specimen one at 0.35 percent of 50 mm long and 0.15 mm thick RSF showed a significant 10% increase in flexure strength [52]. 

Conversely, a slight increase of 4% for concrete was reported at 0.20% for 62 mm long and 0.80 mm thick RSF [54]. It showed that greater improvement in FS can be achieved in the mortar of zero coarse aggregate mixture by using the same amount of RSF. When RSF was increased from 0.35 percent to 0.70 percent in specimen four for the same-size (50 mm long and 0.15 mm thick) RSF, a 13 percent enhancement in FS was detected [52]. In comparison to specimen one, employing about double the amount (0.57 percent by volume fraction) of shorter RSF (21 mm long) resulted in a considerable increase of 69% and 31% in flexure strength of specimens two and three, respectively [68]. A substantial increase in FS was reported when utilizing RSFs that had lengths of 21 mm and 50 mm and diameters of 0.15 mm and 0.20 mm from 0.35% to 0.70%. In addition, significant enhancement in FS was reported by adding 1% to 3% RSF. Within the range of 1% to 3%, a maximum increase of 57% was reported for specimen 11, incorporating moderately sized 2.50% RSF (25 mm in length and 0.26 mm in diameter) [33]. A gradual increase of 31%, 41%, 55%, and 57% was noted in FS at 1%, 1.50%, 2%, and 2.50% RSF (25 mm by 0.26 mm in size), respectively, in the same study [33]. At the same 1.50% RSF, different amounts of increase in FS were reported for specimens seven, eight, and nine, incorporating different sizes of RSF. It can be noted that the longest fibers (50 mm) in specimen nine showed minor improvement in FS as compared to specimens seven and eight, at 1.50% [24]. At 3% of medium-length RSF (31.70 mm length), an improvement of 48% was noticed in FS [67]. A minimum 19% enhancement in FS was reported for the lengthiest RSF (50 mm), specimen six, at 1.05% compared to other specimens incorporating more than 1% content of short- and medium-sized RSFs. This indicated that the short- and medium-size RSF could perform well when used at more than 1% content.

## 6. Correlation between Strength Properties of RSF Concrete

Some standards for plain concrete specify the correlation between compressive strength and flexural or splitting strength. These include the relation between the compressive strength, split tensile strength, and flexure strength specified by the American Concrete Institute (ACI) and British Standards (BS). The equations are given below:

(1) $$\begin{array}{cc} \mathrm{STS}=0.56\;\sqrt{fc^{\prime }} & \;\;\;\;\;\;\;\;\mathrm{ACI}\;\mathrm{committee} \end{array}$$

(2) $$\begin{array}{cc} \mathrm{FS}=0.60\;\sqrt{fc^{\prime }} & \;\;\;\;\;\;\;\;\mathrm{BS-}8110 \end{array}$$

**Figure 19:**
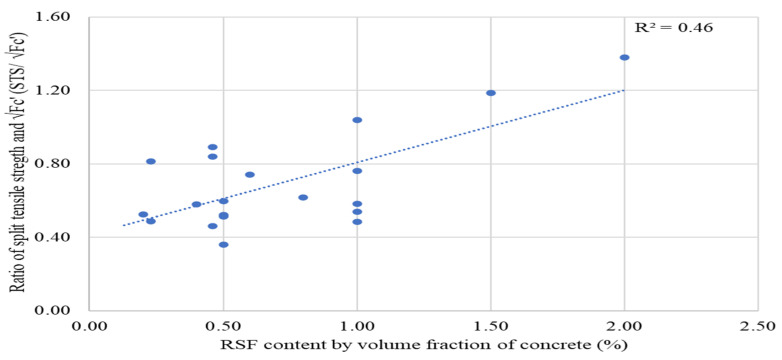
Effect of RSF (0% to 2% by volume fraction of concrete) on the ratio between compressive strength and split tensile strength.

The potential relationship between flexural strength and split tensile strength is shown by Equations (1) and (2). The STS/√(*fc′*) ratio increases linearly by adding fiber to 2%, as seen in Figure 19, indicating that the RSF helps improve tensile strength. Furthermore, the STS/√(*fc′)* ratio also has a rising trend with an increase in the content of RSF. This showed that the ratio recommended for compressive strength and split tensile strength of plain concrete are not valid for RSF concrete and need to be modified accordingly.

**Figure 20:**
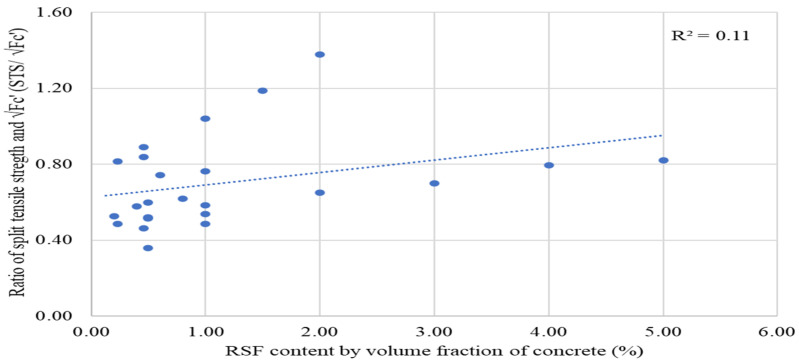
Effect of RSF (0% to 5%) on the ratio between compressive strength and split tensile strength.

Better compliance is supported by the moderate value (0.46) of the coefficient of regression (R^2^) to 2% RSF. However, the regression coefficient (R^2^) is lowered when the trend line for fiber content is drawn to 5% RSF as shown in Figure 20. The compliance between RSF content by volume fraction of concrete and STS/√(fc′) ratio is lowered when the trend line is extended to the results of one study, which reported STSs for inclusion of 3%, 4%, and 5% of RSF [62].

Therefore, a more reliable comparison between the fiber content above 2% and the STS/√(*fc*′) ratio can be made when more studies on FS of concrete incorporate more than 2% RSF. Therefore, the average value of the STS/√(*fc*′) ratio for 0.20% to 2% RSF is calculated using currently available data in Table 14. 

The average STS/√(*fc*′) ratio value is 0.67, which is more significant than the proposed ratio for plain concrete. Therefore, a more reliable ratio between split tensile strength can be obtained in the future by using a large amount of data related to the experimental results of the concrete’s compressive strength and split tensile strength.

**Figure 21:**
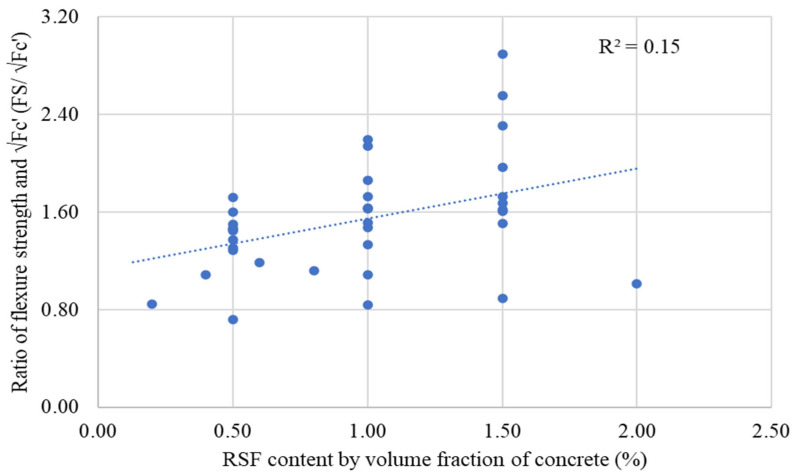
Effect of RSF on the ratio between compressive strength and flexure strength.

The relation between the FS/√(*fc*′) ratio and RSF content of concrete specimens incorporating 0.20% to 2% RSF by volume fraction of concrete is shown in Figure 21. The FS/√(*fc*′) ratio rises with fiber, showing that the RSF helps improve flexural strength. Furthermore, the FS/√(*fc*′) ratio has rises in line with the growth in RSF content. This demonstrated that the plain concrete ratios for compressive and flexure strength are invalid for RSF concrete and should be changed accordingly. A linear trend of increase in the ratio of FS/√(*fc*′) is noted for an increase in the dose of the RSF content by volume fraction of concrete. 

The regression coefficient shows less correlation between the ratio of FS/√(*fc*′) and the content of the RSF due to a low regression coefficient (R^2^) of 0.15; however, it supported that the FS/√(*fc*′) ratio does not follow the same relation as the plain concrete. Therefore, more experimental data are required to formulate a unique FS/√(*fc*′) ratio for RSF concrete. It can be concluded that the existing experimental data are insufficient to conclude a reliable value for the FS/√(*fc′)* ratio. Therefore, more data are required to determine a reliable FS/√(*fc*′) ratio. However, an average value for the FS/√(*fc*′) ratio is premeditated for the currently available data for RSF concrete containing 0.20% to 2% RSF, as shown in Table 15.

The average FS/√(*fc′*) ratio is 1.39, higher than the recommended plain concrete ratio. In the future, a more trustworthy ratio between flexure strength and compressive strength of concrete can be obtained by analyzing vast amounts of data linked to experimental results of the compressive and flexure strength of concrete.

Furthermore, the diameter, length, and aspect ratio of the RSF also need to be considered. Because the properties of the RSF have a significant influence on the mechanical properties of the concrete, their influence cannot be ignored. Therefore, extensive research is required to enhance the database and obtain reliable relations among the various mechanical properties of RSF concrete.

## 7. Possible Applications

The review of previous studies showed that recycled steel fibers of waste tires (RSF) have the potential to enhance the strength properties of concrete. It is expected that RSFs can replace industrial steel fibers (ISF) if uniformity is maintained in the RSFs by using more advanced procedures for their extraction and cutting. The possible applications of RSF concrete can be concluded based on the available data related to experimental testing of RSF concrete. Therefore, the applications of RSF concrete for various civil engineering structures and fields are discussed in comparison to other types of fibers in the section below.

### 7.1. Application of RSF for Rigid Pavements

The controlling parameters for the design of rigid pavements, including street pavements and concrete roads, are flexure and compressive strength. According to the AASHTO (1993) equation [69], AASHTOWare Pavement ME Design software [70], the American Concrete Pavement Association (ACPA) pavement design software, StreetPave [71], and Jiang et al. [72], the two main variables that affect the design of a concrete pavement and its thickness are the flexure strength (FS) and modulus of elasticity (depending on CS). Following these recommendations, hair fiber reinforced concrete (HFRC) was proposed as a less expensive and more effective alternative for concrete roadways due to increases of 12.4% and 16.2% in CS and FS, respectively, compared to plain concrete [73]. On the other hand, RF-incorporated concrete showed an increase of 78% and 162% in CS and FS, respectively, compared to PC, as reported by Younis [58] and Köroğlu [62] correspondingly. Therefore, it is expected that RSF concrete can perform much better than HFRC in reducing the thickness of concrete at less cost. In addition, the tendency of RSF to improve the post-cracking behavior and post-crack energy absorption is also expected to be more due to its higher tensile strength than hair fibers. The tensile strength of the RSF is also higher than natural fibers such as jute and bagasse fibers.

Moreover, the RSF can be an excellent replacement for costly artificial fibers like nylon and glass. Thus, RF-incorporated concrete has the potential to replace other types of costly and less durable natural fibers for achieving better pre-crack and post-crack properties of concrete for achieving long-term durability. Therefore, RFs are likely the best for concrete roads and street pavements. However, an in-depth analysis of the likelihood of RSF replacing the natural and other types of fibers still needs more research; the durability in particular needs to be tested. 

### 7.2. Application of RSF for Hydraulic Structures

The cracking rate in hydraulic concrete structures, such as canal lining, may be influenced by various variables, including shrinkage, water absorption, permeability, differential settlement, and tensile strength [74]. If the tensile stresses caused by shrinkage are less than the tensile strength of concrete, cracking from shrinkage can be avoided. This demonstrates that concrete’s tensile strength is essential for preventing shrinkage cracks. The other possible reason for the decrease in serviceability of the concrete lining of drains and canals is the differential settlement of the concrete structure. The differential settlement causes bending stresses; thus, higher flexural strength is required to control the differential settlements. Combining all these factors, fiber-reinforced concrete is suggested as the best solution for controlling the cracking rate in thin hydraulic structures compared to plain concrete. Many authors suggested polypropylene and nylon fibers as an effective solution for increasing the long-term durability of canal lining and hydraulic structures. For example, Zia and Ali [17] suggested polypropylene fiber-reinforced concrete (PPFRC) as an effective material for controlling the rate of cracking in canal lining. The authors noticed an improvement of 1%, 5%, and 34% in CS, STS, and FS of PPFRC compared to PC. On the other hand, the PPFRC was also suggested to be effective in increasing the long-term durability of hydraulic structures [75].

On the other hand, according to the available database, the possible increase in CS, STS, and FS of RSF-incorporated concrete is 78%, 149%, and 162%, respectively, reported by Younis [58], Abdul Awal et al. [53], and Köroğlu [62], correspondingly. Therefore, the strength properties of the RSF concrete improved more compared to PPFRC. This justifies the capability of RSF-incorporated concrete to replace PPFRC as a cheap solution for improving the durability of hydraulic structures.

### 7.3. Application of RSF for Reinforced Concrete Structures

The beam-column joints of reinforced concrete (RC) structures are very important. Various authors suggest that the failure of the building starts from joints [76,77]. Therefore, to increase the sustainability of RC frame structures during extreme events such as earthquakes, it is essential to enhance the material’s compressive strength to be used in the joint core. In addition, it could help enhance the shear strength of the joint core, which is the only action to counter the effect of lateral loads on joints. The shear strength of the joints is directly related to the compression strength of the RC beam-column joints [78].

To increase the beam-column joints’ performance, many materials have been tested to check their suitability for an increase in joint shear strength. Adibi et al. [79] employed steel angles prestressed by crossties to retrofit exterior concrete beam-column connections with hook-ended plain bars seismically. Cosgun et al. [80] examined the RC frames’ experimental behavior with FRP strengthening to check FRP’s suitability for avoiding joint failure. D’Ayala et al. [81] also evaluated the application of FRP strengthening for plain bar-reinforced interior joints. Ultra-high performance hybrid fiber-reinforced concrete was also used to increase RC beam-column joints’ performance [82]. All these studies aimed to increase the compressive strength of concrete to achieve the maximum possible joint shear strength. The current data showed that by using RSF, the CS of normal concrete could be improved by 78%. Therefore, RSF concrete can be a good choice for improving the seismic behavior of beam-column joints at low cost. 

## 8. Conclusions and Recommendations

A complete review of the mechanical properties of recycled steel fibers of waste tires (RSF), concrete, and mortars were presented in this paper. First, RSF’s contribution to hardened concrete and mortar qualities and long-term development were contrasted with plain ones. A vast number of papers have been studied in this respect. The effect of various doses of RSF on the compressive, tensile, and flexural strengths of the concrete and mortars were discussed. A critical review of previous papers was also given. The mistakes noted in the previous review papers were avoided here. An extensive database was used in this study. Detailed studies regarding the statistical analysis of RSFs used, properties of RSFs, and the percent increase or decrease in the strength properties of the RSF composite compared with plain concrete or mortar were reported. In addition, a detailed summary of the possible increase or decrease in compressive strengths, split tensile strength, and flexure strength reported in the previous specific dose of RSF were demonstrated. The effect of different parameters (length and diameter) of RSFs on various strength properties of concrete and mortars was also discussed. The following conclusions can be made from a thorough review of research data related to the strength properties of RSF concrete:

- A maximum enhancement of 29% was reported in compressive strength (CS) at 0.40% RSF (62 mm long and 0.80 mm thick) added by the volume fraction of concrete. A maximum enhancement of 78% in CS at 4% RSF (29 mm long and 0.20 mm thick) by weight fraction of concrete was reported. The most significant improvement in CS was reported at 50 kg/m^3^ RSF (17.50 mm long and 0.25 mm thick) by adding RSFs in kg/m^3^ to concrete.
- Split tensile strength (STS) was increased maximally by up to 149% when two percent RSF (62 mm long) was added to concrete by volume fraction. The highest increase of 96% in STS at 6% RSF (29 mm in length and 0.20 mm in diameter) for RSF by weight fraction of concrete was reported. The most significant improvement of 43% in STS was reported at 50 kg/m^3^ RSF (17.50 mm long and 0.25 mm thick) when adding RSFs in kg/m^3^ to concrete.
- The maximum increase in flexure strength (FS) was up to 157% when adding 3% RSF (45 mm long and 0.245 mm thick) into the concrete by volume fraction. An increase of 457% and 429% in FS was reported at 4% and 5% of RSF. However, such a high increase in FS needs to be explored and verified by additional research. At 6% RSF (29 mm in length and 0.20 mm in diameter) added to concrete by its weight proportion, the maximum increase in FS was 162 percent. The greatest improvement in FS was 68% when adding 40 kg/m^3^ RSFs (50 mm long and 0.60 mm thick) to concrete.
- The size of RSFs have a significant impact on the concrete’s CS. A considerable rise in CS was observed by many researchers for the same proportion of lengthier RSFs.
- It was found that small RSFs work better than large RSFs for increasing the STS of concrete. In comparison, longer RSFs were more effective than smaller RSFs in improving the FS of concrete.
- The most significant increases of 46%, 50.6%, and 69%, were reported in CS, STS, and FS of cement mortars (zero coarse aggregate mixture), adding 1.50% RSF (50 mm long and 0.15 mm thick), 0.02% RSF (50 mm long and 0.15 mm thick), and 0.57% RSF (21 mm lengthy and 0.20 mm thick), respectively, by volume fraction of mortar.
- In mortar, the low proportion of lengthy RFs worked effectively in improving CS. It is noted that even at a low dose of RFs for mortar, a sizable increment in CS was observed. In contrast to RF mortar, the rise in CS for RF concrete was minimal. Compared to other RFs, medium-size (20 to 35 mm long) RFs performed better in improving their FS. Therefore, it is vital to introduce advanced techniques to improve the RSF mixes’ homogeneity and the dispersion of RSF within the mix of mortar and concrete.
- The increase or decrease of RSF on the concrete’s CS is mostly no more than 48%. However, opposite to the low volume of fiber concrete, if the fiber content was controlled at higher than moderate volume (1% to 2% by volume fraction of concrete), the improvement showed a slightly dropping trend with RSF. The impact of RSF on the compressive strength of various cement-based mixtures, especially mortar, is yet unknown; more research is needed to fully comprehend the fiber’s interaction with the matrix and its behavior under compressive loads. However, unlike mortar, experimental results reveal that maintaining the RSF fiber content at lower than 1% of the volume fraction of concrete slightly improves compressive strength capacity.
- For similar content of RSF, mechanical strengths can differ when using different lengths of RSF. It was observed that different authors noted variations in compressive, split tensile, and flexure strengths for the different lengths of RSF used in the same dose of RSFs. Therefore, for optimizing RSF’s content for any mix, dimensional parameters, especially the length of RSF, also need to be considered.

An in-depth investigation of the hardened properties of concrete for various sizes of recycled steel fibers from discarded tires is still missing. Therefore, an extensive campaign of studies is required to evaluate different sizes of RFs at the same content levels. Nevertheless, it may help create a dataset that can assist in obtaining refined results for different applications. Evaluating the effect of more than 2% RSF (added by volume fraction of composites) on concrete and mortar strength properties is recommended. The existing database is insufficient for concluding the effect of using various sizes of RSF in more than 2% by volume fraction of concrete. The influence of using high doses (more than 2% by volume fraction of concrete) of small-sized RSFs needs to be explored.

## Figures and Tables

**Table 1:** Characteristics of the raw steel fibers recovered from waste tires used in cement composites.

Authors	Fiber Properties		
	Average Length	Average Diameter	Tensile Strength
	(mm)	(mm)	(MPa)
Gul et al. [46]	100.16	0.939	996.15
	7.62	0.939	996.15
Rossli and Ibrahim [37]	60	0.80	-
Siraj and Kedir [47]	20	0.89	970.2
	40	0.89	970.2
	60	0.89	970.2
Leone et al. [41]	13.94	0.25	-
Caggiano et al. [48]	26.17	0.25	-
Pawelska-Mazur and Kaszynska [21]	17.50	0.25	2200
Sengul [49]	50	0.3	-
	50	0.6	1330
	50	1.4	1160
Frazão et al. [50]	23	0.22	2570
Najim et al. [51]	50	-	781.3
Vistos et al. [42]	12	0.27	2235
Mastali et al. [24]	50	0.15	1150
Mastali et al. [52]	50	0.15	1150
Samarakoon et al. [25]	37	0.42	870
Peng et al. [28]	30	1	1900
	35	1	1900
Abdul Awal et al. [53]	30	-	1030
Skarzynski and Suchorzewski [54]	26.17	0.25	-
Aiello et al. [44]	26	0.258	2377
Chen et al. [55]	7.3	0.22	2165
Dorr et al. [56]	93.6	1.17	1032.35
Suleman et al. [57]	45	0.35	1300
Younis [58]	29	0.2	2000
Graeff et al. [59]	13	0.2	2000
Shah et al. [60]	31	-	2000
Akhtar et al. [61]	30	1.1	1900
Köroğlu [62]	45	0.245	2134
Centonze et al. [63]	31.4	0.24	-
Leone et al. [64]	31.4	0.24	-
Groli et al. [65]	21	-	-
Maximum value	7.3	0.15	781.3
Minimum value	100.16	1.4	2570

**Table 2:** Results of compressive strength’s percent comparison and fiber properties of the concrete encompassing less than 1% of RSF by its volume fraction.

Authors	Specimen Number in Figure 4	Fiber Properties			Compressive Strength	
		Content	Length	Diameter		
Fiber Less Than 0.30%		% ^a^	mm	mm	MPa	% ^b^
Aiello et al. [44]	1 (1% Sp)	0.13	26.0	0.258	40.4	103
	2 (1% Sp)	0.15	26.0	0.258	32.2	82
	3 (1% Sp)	0.19	26.0	0.258	34.9	89
	4 (1.20% Sp) *	0.19	26.0	0.258	33.4	85
Rossli and Ibrahim [37]	5 (0.002 Sp)	0.20	62.00	0.8	52.0	113
Aiello et al. [44] (Planetary mixer)	6 (1.12%)	0.23	26.0	0.258	38.5	120
Centonze et al. [63] (Planetary mixer)	7	0.23	31.4	0.24	39.0	123
Aiello et al. [44] (Normal concrete mixure)	8 (1.20% Sp)	0.26	26.0	0.258	34.6	89
	9 (1.37% Sp)	0.26	26.0	0.258	43.7	112
Leone et al. [64]	10	0.3	31.4	0.24	35.71	105
Average		0.21	30.68	0.31		**102**
0.40% to 0.50% fiber						
Rossli and Ibrahim [37]	11	0.40	62.00	0.8	59.2	129
Leone et al. [41]	12	0.46	13.94	0.25	32.6	97
Centonze et al. [63] (Planetary mixer)	13	0.46	31.4	0.24	39.7	125
Aiello et al. [44] (Planetary mixer)	14 (1.12% Sp)	0.46	26.00	0.258	37.6	117
Groli et al. [65]	15	0.5	21	-	36.4	97
Siraj and Kedir [47] (Mix-I, C-25)	16	0.50	20.00	0.89	28.0	100
	17	0.50	40.00	0.89	28.2	101
	18	0.50	60.00	0.89	28.2	101
Siraj and Kedir [47] (Mix-II, C-40)	19	0.50	20.00	0.89	40.9	100
	20	0.50	40.00	0.89	41.5	101
	21	0.50	60.00	0.89	41.6	101
Siraj and Kedir [47] (Mix-III, C-60)	22	0.50	20.00	0.89	59.0	101
	23	0.50	40.00	0.89	60.5	104
	24	0.50	60.00	0.89	56.3	96
Vistos et al. [42]	25	0.50	12.00	0.27	37.4	102
Samarakoon et al. [25]	26	0.50	37.00	0.42	30.6	105
Skarżyński and Suchorzewski [54]	27	0.50	26.17	0.25	52.4	122
Chen et al. [55]	28	0.50	7.30	0.22	51.6	103
Dorr et al. [56]	29	0.50	93.60	1.17	31.3	111
Average		0.49	36.34	0.66		106
0.60% to 0.80% fiber						
Rossli and Ibrahim [37]	30	0.60	62.00	0.8	34.5	75
Caggiano et al. [48]	31	0.75	26.17	0.25	22.5	102
Chen et al. [55]	32	0.75	7.30	0.22	57.6	115
Rossli and Ibrahim [37]	33	0.80	62.00	0.8	40.1	87
Average		0.73	39.37	0.52		94.92

* Sp is the quantity of superplasticizer added to the mix. ^a^ content of fibers added by volume fraction of concrete, ^b^ percent increase or decrease in strength of RSF concrete w.r.t plain concrete (100%).

**Table 3:** Results of compressive strength’s percent comparison and fiber properties of the concrete having 1% or greater than 1% RSF by its volume fraction.

Authors	Specimen Number in Figure 4	Fiber Properties			Compressive Strength	
		Content	Length	Diameter		
1% Fiber Content		% ^a^	mm	mm	MPa	% ^b^
Rossli and Ibrahim [37]	34	1.00	62.00	0.8	42.3	92
Groli et al. [65]	35	1	21	-	37.5	100
Köroğlu [62]	36	1	45	0.245	36.26	99
Siraj and Kedir [47] (Mix-I, C-25)	37	1.00	20.00	0.89	27.9	100
	38	1.00	40.00	0.89	29.1	104
	39	1.00	60.00	0.89	27.7	99
Siraj and Kedir [47] Mix-II, C-40	40	1.00	20.00	0.89	41.3	101
	41	1.00	40.00	0.89	43.7	106
	42	1.00	60.00	0.89	41.1	100
Siraj and Kedir [47] Mix-III, C-60	43	1.00	20.00	0.89	27.9	100
	44	1.00	40.00	0.89	29.1	104
	45	1.00	60.00	0.89	27.7	99
Samarakoon et al. [25]	46	1.00	37.00	0.42	32.9	113
Abdul Awal et al. [53]	47	1.00	30.00	-	58.2	104
Chen et al. [55]	48	1.00	7.30	0.22	56.3	113
Average		1	37	1		102
More than 1.25% Fiber						
Chen et al. [55]	49	1.25	7.30	0.22	52.7	105
Siraj and Kedir [47] (Mix-I, C-25)	50	1.50	20.00	0.89	29.0	103
	51	1.50	40.00	0.89	31.4	112
	52	1.50	60.00	0.89	28.1	100
Siraj and Kedir [47] Mix-II, C-40	53	1.50	20.00	0.89	41.8	102
	54	1.50	40.00	0.89	43.9	107
	55	1.50	60.00	0.89	42.0	102
Siraj and Kedir [47] Mix-III, C-60	56	1.50	20.00	0.89	62.4	107
	57	1.50	40.00	0.89	59.2	101
	58	1.50	60.00	0.89	61.0	104
Abdul Awal et al. [53]	59	1.50	30.00	-	59.0	106
	60	2.00	30.00	-	58.7	105
Köroğlu [62]	61	2	45	0.245	43.1	118
	62	3	45	0.245	44.2	121
	63	4	45	0.245	46.2	127
	64	5	45	0.245	43.8	120
Average		2.02	38	1		109

^a^ content of fibers added by volume fraction of concrete, ^b^ percent increase or decrease in strength of RSF concrete w.r.t plain concrete (100%).

**Table 4:** Results of compressive strength’s percent comparison and fiber properties of the concrete with RSF by its weight.

Author	Specimen Number in Figure 6	Fiber Properties			Compressive Strength	
		Content	Length	Diameter		
0.20% to 0.75% Fiber		% ^a^	mm	mm	MPa	% ^b^
Shah et al. [60]	1	0.1	31.00	-	56.7	102
	2	0.25	31.00	-	58.3	105
Akhtar et al. [61]	3	0.25	30.00	1.10	78.0	104
Shah et al. [60]	4	0.5	31.00	-	54.0	97
Akhtar et al. [61]	5	0.5	30.00	1.10	79.5	106
Shah et al. [60]	6	0.75	31.00	-	52.5	95
Average		0.39	30.67	1.10	63.2	101.5
1% to 2% Fiber						
Shah et al. [60]	7	1	31.00	-	49.7	90
Akhtar et al. [61]	8	1	30.00	1.10	77.0	103
Gul et al. [46]	9	2	100.16	0.94	35.9	127
	10	2	7.62	0.94	35.5	126
Younis [58]	11	2	29.00	0.20	47.6	168
Graeff et al. [59]	12	2	13.00	0.20	61.1	104
Average		1.67	35.13	0.68	51.1	119.5
2.5% to 3.5% Fiber						
Gul et al. [46]	13	2.5	100.16	0.94	37.9	134
	14	2.5	7.62	0.94	38.6	137
	15	3	100.16	0.94	27.9	99
	16	3	7.62	0.94	32.4	115
	17	3.5	100.16	0.94	25.5	90
	18	3.5	7.62	0.94	26.9	95
Average		3.00	53.89	0.94	31.5	111.5
4% to 6% Fiber						
Gul et al. [46]	19	4	100.16	0.94	23.4	83
	20	4	7.62	0.94	26.2	93
Younis [58]	21	4	29.00	0.20	50.4	178
	22	6	29.00	0.20	47.2	167
Graeff et al. [59]	23	6	13.00	0.20	71.4	122
Average		4.80	35.76	0.50	43.7	128.5

^a^ content of fibers added by weight fraction of concrete, ^b^ percent increase or decrease in strength of RSF concrete w.r.t plain concrete (100%).

**Table 5:** Results of compressive strength’s percent comparison and fiber properties of the concrete with RSF in kg/m^3^.

Author	Specimen No. in Figure 7	Fiber Properties			Compressive Strength	
		Content	Length	Diameter		
5 to 30 kg/m^3^		kg/m^3^	mm	mm	MPa	% ^a^
Sengul [49]	1	5	52.00	0.30	69.50	100
	2	10	52.00	0.30	65.80	95
	3	10	50.00	0.60	64.10	92
	4	15	52.00	0.30	70.60	102
	5	20	50.00	0.60	64.70	93
	6	20	50.00	1.40	68.70	99
	7	30	50.00	0.60	71.50	103
Average		15.71	50.86	0.59		98
40 to 60 kg/m^3^						
Sengul [49]	8	40	50.00	0.60	75.30	109
	9	40	50.00	1.40	69.10	100
Pawelska-Mazur and Kaszynska [21]	10	50	17.50	0.25	52.40	122
Sengul [49]	11	60	50.00	1.40	63.60	92
Peng et al. [28]	12	60	30.00	1.00	154.30	114
	13	60	35.00	1.00	141.30	105
Average		51.67	38.75	0.94	-	107

^a^ percent increase or decrease in strength of RSF concrete w.r.t plain concrete (100%).

**Table 6:** Results of split tensile strength’s percent comparison and fiber properties of the concrete incorporating RSF by its volume fraction.

Author	Specimen No. in Figure 8	Fiber Properties			Split Tensile Strength	
		Content	Length	Diameter		
0.20% to 0.80% Fiber Content		% ^a^	mm	mm	MPa	% ^b^
Rossli and Ibrahim [37]	1	0.20	62.00	0.8	3.39	87
Aiello et al. [44]	2	0.23	26.00	0.258	2.70	116
Leone et al. [64]	3	0.30	31.40	0.24	4.73	104
Rossli and Ibrahim [37]	4	0.40	62.00	0.8	3.99	103
Leone et al. [41]	5	0.46	13.94	0.25	4.55	90
Aiello et al. [44]	6	0.46	26.00	0.258	2.54	109
Samarakoon et al. [25]	7	0.50	37.00	0.42	2.58	118
Skarżyński and Suchorzewski [54]	8	0.50	26.17	0.25	3.87	143
Dorr et al. [56]	9	0.50	93.60	1.17	1.80	86
Groli et al. [65]	10	0.50	21.00	-	3.1	74
Rossli and Ibrahim [37]	11	0.60	62.00	0.8	3.90	100
	12	0.80	62.00	0.8	3.50	90
Average		0.45	43.59	0.55		102
1% to 2% Fiber Content						
Groli et al. [65]	13	1	21.00	-	3.3	79
Köroğlu [62]	14	1	45.00	0.245	3.14	116
Rossli and Ibrahim [37]	15	1	62.00	0.8	4.44	114
Samarakoon et al. [25]	16	1	37.00	0.42	2.49	114
Abdul Awal et al. [53]	17	1	30.00	-	7.10	187
	18	1.5	30.00	-	8.15	214
	19	2	30.00	-	9.45	249
Köroğlu [62]	20	2	45.00	0.245	3.82	141
	21	3	45.00	0.245	4.16	154
	22	4	45.00	0.245	4.84	178.6
	23	5	45.00	0.245	4.86	179.3
Average		2.05	39.55	0.35	5.07	157

^a^ content of fibers added by volume fraction of concrete, ^b^ percent increase or decrease in strength of RSF concrete w.r.t plain concrete (100%).

**Table 7:** Results of split tensile strength’s percent comparison and fiber properties of the concrete incorporating RSF by its weight fraction.

Author	Specimen Number In Figure 10	Fiber Properties			Split Tensile Strength	
		Content	Length	Diameter		
0.25% to 1% Fiber Content		% ^a^	mm	mm	MPa	% ^b^
Akhtar et al. [61]	1	0.25	30	1.1	6.10	135.6
	2	0.5	30	1.1	6.80	151.1
	3	1	30	1.1	7.20	160.0
Average		0.58	30.00	1.10		148.89
2% to 3.5% Fiber Content						
Gul et al. [46]	4	2	100.16	0.94	3.45	147.1
	5	2	7.62	0.94	3.38	144.1
Younis [58]	6	2	29.00	0.20	4.20	179.1
Gul et al. [46]	7	2.5	100.16	0.94	3.52	150.0
	8	2.5	7.62	0.94	3.45	147.1
	9	3	100.16	0.94	3.59	152.9
	10	3	7.62	0.94	3.52	150.0
	11	3.5	100.16	0.94	3.99	170.3
	12	3.5	7.62	0.94	3.70	157.9
Average		2.67	51.12	0.86		155.39
4% to 6% Fiber Content						
Gul et al. [46]	13	4	100.16	0.94	2.90	123.5
	14	4	7.62	0.94	2.55	108.8
Younis [58]	15	4	29.00	0.20	4.60	196.2
	16	6	29.00	0.20	4.60	196.2
Average		4.50	41.45	0.57	-	156.18

^a^ content of fibers added by weight fraction of concrete, ^b^ percent increase or decrease in strength of RSF concrete w.r.t plain concrete (100%).

**Table 8:** Results of split tensile strength’s percent comparison and fiber properties of the concrete incorporating RSF in kg/m^3^.

Author	Specimen No. in Figure 11	Fiber Properties			Split Tensile Strength	
		Content	Length	Diameter		
5 to 20 kg/m^3^		kg/m^3^	mm	mm	MPa	% ^a^
Sengul [49]	1	5	52.00	0.30	5.60	84
	2	10	52.00	0.30	6.50	97
	3	10	50.00	0.60	6.00	90
	4	15	52.00	0.30	6.60	99
	5	20	50.00	0.60	7.20	107
	6	20	50.00	1.40	6.80	101
	7	30	50.00	0.60	7.90	118
Average		15.71	50.86	0.59	-	99
20 to 60 kg/m^3^						
Sengul [49]	8	40	50.00	0.60	9.00	134
	9	40	50.00	1.40	7.20	107
Pawelska-Mazur and Kaszynska [21]	10	50	17.50	0.25	3.87	143
Sengul [49]	11	60	50.00	1.40	7.00	104
Peng et al. [28]	12	60	30.00	1.00	8.75	127
	13	60	35.00	1.00	9.39	136
Average		51.67	38.75	0.94	-	125

^a^ percent increase or decrease in strength of RSF concrete w.r.t plain concrete (100%).

**Table 9:** Results of flexure strength’s percent comparison and fiber properties of the concrete incorporating RSF by its volume fraction.

Author	Specimen Number in Figure 12	Fiber Properties			Flexure Strength	
		Content	Length	Diameter		
0.20% to 0.80% Fiber Content		% ^a^	mm	mm	MPa	% ^b^
Rossli and Ibrahim [37]	1	0.2	62	0.80	5.45	102
	2	0.4	62	0.80	7.49	140
Siraj and Kedir [47] Mix-I, C-25	3	0.5	20	0.89	6.84	111
	4	0.5	40	0.89	6.96	113
	5	0.5	60	0.89	8.19	133
Siraj and Kedir [47] Mix-I, C-40	6	0.5	20	0.89	8.34	108
	7	0.5	40	0.89	8.64	112
	8	0.5	60	0.89	9.24	120
Siraj and Kedir [47] Mix-I, C-60	9	0.5	20	0.89	8.85	101
	10	0.5	40	0.89	9.09	104
	11	0.5	60	0.89	9.21	105
Skarżyński and Suchorzewski [54]	12	0.5	26.17	0.25	4.67	130
Rossli and Ibrahim [37]	13	0.60	62	0.80	6.24	117
	14	0.80	62	0.80	6.36	119
Average		0.50	45.30	0.82		115.24
1% to 2% Fiber Content						
Rossli and Ibrahim [37]	15	1	62	0.80	6.321	118
Köroğlu [62]	16	1	45	0.245	3.60	171
Siraj and Kedir [47] Mix-I, C-25	17	1	20	0.89	7.17	116
	18	1	40	0.89	7.89	128
	19	1	60	0.89	10.08	163
Siraj and Kedir [47] Mix-I, C-40	20	1	20	0.89	8.46	110
	21	1	40	0.89	9.60	124
	22	1	60	0.89	12.57	163
Siraj and Kedir [47] Mix-I, C-60	23	1	20	0.89	9.30	107
	24	1	40	0.89	12.00	138
	25	1	60	0.89	12.84	147
Abdul Awal et al. [53]	26	1	30	-	5.75	125
Siraj and Kedir [47] Mix-I, C-25	27	1.5	20	0.89	8.07	131
	28	1.5	40	0.89	8.64	140
	29	1.5	60	0.89	13.71	222
Siraj and Kedir [47] Mix-I, C-40	30	1.5	20	0.89	8.7	113
	31	1.5	40	0.89	9.60	124
	32	1.5	60	0.89	14.79	192
Siraj and Kedir [47] Mix-I, C-60	33	1.5	20	0.89	11.37	130
	34	1.5	40	0.89	13.53	155
	35	1.5	60	0.89	16.14	185
Abdul Awal et al. [53]	36	1.5	30	-	6.15	134
	37	2	30	-	6.95	151
Köroğlu [62]	38	2	45	0.245	3.90	186
Köroğlu [62]	39	3	45	0.245	5.40	257
Köroğlu [62]	40	4	45	0.245	11.70	557
Köroğlu [62]	41	5	45	0.245	11.10	529
Average		1.59	41	0.75		178

^a^ content of fibers added by volume fraction of concrete, ^b^ percent increase or decrease in strength of RSF concrete w.r.t plain concrete (100%).

**Table 10:** Results of flexure strength’s percent comparison and fiber properties of the concrete incorporating RSF by weight fraction.

Author	Specimen Number in Figure 14	Fiber Properties			Flexure Strength	
		Content	Length	Diameter		
0.25% to 1% Fiber Content		% ^a^	mm	mm	MPa	% ^b^
Shah et al. [60]	1	0.10	31.00	-	5.23	116
	2	0.25	31.00	-	5.7	127
Akhtar et al. [61]	3	0.25	30.00	1.10	9.3	124
Shah et al. [60]	4	0.50	31.00	-	6.41	142
Akhtar et al. [61]	5	0.50	30.00	1.10	11.8	157
Shah et al. [60]	6	0.75	31.00	-	5.63	125
	7	1	31.00	-	5.53	123
Akhtar et al. [61]	8	1	30.00	1.10	12.8	171
Average		0.54	30.63	1.10		136
2% to 3.5% Fiber Content						
Akhtar et al. [46]	9	2	100.16	0.94	7.17	179
	10	2	7.62	0.94	6.34	159
Younis [58]	11	2	29.00	0.20	4.60	100
Graeff et al. [59]	12	2	13.00	0.20	6.47	135
Gul et al. [46]	13	2.5	100.16	0.94	7.72	193
	14	2.5	7.62	0.94	6.62	166
	15	3	100.16	0.94	8.97	224
	16	3	7.62	0.94	7.45	186
	17	3.5	100.16	0.94	10.5	262
	18	3.5	7.62	0.94	8.14	203
Average		2.60	47.31	0.79		181
4% to 6% Fiber Content						
Gul et al. [46]	19	4	100.16	0.94	9.1	228
	20	4	7.62	0.94	8.9	222
Younis [58]	21	4	29.00	0.20	5	109
	22	6	29.00	0.20	4.8	104
Graeff et al. [59]	23	6	13	0.2	7.11	148
Average		4.80	35.76	0.50		162

^a^ content of fibers added by weight fraction of concrete, ^b^ percent increase or decrease in strength of RSF concrete w.r.t plain concrete (100%).

**Table 11:** Results of flexure strength’s percent comparison and fiber properties of the concrete incorporating RSF in kg/m^3^.

Author	Specimen Number in Figure 15	Fiber Properties			Flexure Strength	
		Content	Length	Diameter		
5 to 20 kg/m^3^		kg/m^3^	mm	mm	MPa	% ^a^
Sengul [49]	1	5	52.00	0.30	7.40	132
	2	10	52.00	0.30	5.10	91
	3	10	50.00	0.60	5.70	102
	4	15	52.00	0.30	7.20	129
	5	20	50.00	0.60	5.40	96
	6	20	50.00	1.40	6.80	121
Average	-	13.33	51.00	0.58	-	112
30 to 60 kg/m^3^						
Sengul [49]	7	30	50.00	0.60	7.70	138
	8	40	50.00	0.60	9.40	168
	9	40	50.00	1.40	6.70	120
	10	60	50.00	1.40	6.70	120
Average	-	42.5	50.00	1.00	-	136

^a^ percent increase or decrease in strength of RSF concrete w.r.t plain concrete (100%).

**Table 12:** Results of compressive strength’s percentage comparison and fiber properties of the cement mortar incorporating RSF by volume fraction.

Author	Specimen Number in Figure 16	Fiber Properties			Compressive Strength	
		Content	Length	Diameter		
0.10% ^b^ to 1% ^b^ Fiber		% ^b^	mm	mm	MPa	% ^c^
Shi et al. [66]	1	0.01	14.9	0.32	44.1	99.3
	2	0.02	14.9	0.32	48.9	110.3
Mastali and Dalvand [52]	3	0.35	50	0.15	57.3	109.0
Al-musawi et al. [32] (0.21 Sp) ^d^	4	0.57	21	0.20	43.0	111.7
Al-musawi et al. [32] (0.60 Sp) ^d^	5	0.57	21	0.20	47.0	135.1
Mastali and Dalvand [52]	6	0.70	50	0.15	61.7	117.4
Average		0.37	28.63	0.22		113.8
1% ^b^ to 3% ^b^ Fiber						
Dehghanpour and Yılmaz [33]	7	1.00	25.00	0.26	62.7	109.8
Mastali and Dalvand [52]	8	1.05	50.00	0.15	65.8	125.2
Dehghanpour and Yılmaz [33]	9	1.50	25.00	0.26	66.6	116.6
Zamanzadeh et al. [67]	10	1.50	31.70	0.92	21.2	95.5
Mastali et al. [24]	11	1.50	50.00	0.15	73.0	146.0
Dehghanpour and Yılmaz [33]	12	2.00	25.00	0.26	70.5	123.3
	13	2.50	25.00	0.26	64.6	112.9
Zamanzadeh et al. [67]	14	3.00	31.70	0.92	22.1	99.5
Average		1.76	32.93	0.40		116.1

^b^ content of fibers added by volume fraction of mortar, ^c^ percent increase or decrease in strength of RSF mortar with regard to plain mortar (100%). ^d^ Percent quantity of superplasticizer added to the mix by cement mass.

**Table 13:** Results of flexure strength’s percent comparison and fiber properties of the cement mortar incorporating RSF by volume fraction.

Author	Specimen Number in Figure 18	Fiber Properties			Flexure Strength	
		Content	Length	Diameter		
0.35% ^b^ to 0.70% ^b^ Fiber		% ^b^	mm	mm	MPa	% ^c^
Mastali and Dalvand [52]	1	0.35	50	0.15	4.75	110
Al-musawi et al. [68]	2	0.57	21	0.20	8.60	169
	3	0.57	21	0.20	5.50	131
Mastali and Dalvand [52]	4	0.70	50	0.15	4.85	113
Average		0.55	35.50	0.18		131
1% ^b^ to 3% ^b^ Fiber						
Dehghanpour and Yılmaz [33]	5	1.00	25.00	0.26	6.83	131
Mastali and Dalvand [52]	6	1.05	50.00	0.15	5.15	119
Dehghanpour and Yılmaz [33]	7	1.50	25.00	0.26	7.34	141
Zamanzadeh et al. [67]	8	1.50	31.70	0.92	8.43	141
Mastali et al. [24]	9	1.50	50.00	0.15	8.20	125
Dehghanpour and Yılmaz [33]	10	2.00	25.00	0.26	8.09	155
	11	2.50	25.00	0.26	8.19	157
Zamanzadeh et al. [67]	12	3.00	31.70	0.92	8.88	148
Average		1.76	32.93	0.40		140

^b^ content of fibers added by volume fraction of mortar, ^c^ percent increase or decrease in strength of RSF mortar with regard to plain mortar (100%).

**Table 14:** The ratio between compressive strength and split tensile strength for RSF concrete.

Author	Fiber	***fc***′	**√*fc***′	STS	STS**/√(*fc***′**)**
	% ^a^	MPa		MPa	
Rossli and Ibrahim [37]	0.20	51.99	7.21	3.39	0.47
	0.40	59.17	7.69	3.99	0.52
	0.60	34.50	5.87	3.90	0.66
	0.80	40.13	6.33	3.50	0.55
	1.00	28.05	5.30	4.44	0.84
M. Leone et al. [41]	0.46	22.00	4.69	4.55	0.97
Samarakoon et al. [25]	0.50	30.60	5.53	2.58	0.47
	1.00	55.85	7.47	2.49	0.33
Abdul Awal et al. [53]	1.00	58.20	7.63	7.10	0.93
	1.50	59.00	7.68	8.15	1.06
	2.00	42.91	6.55	9.45	1.44
Skarżyński and Suchorzewski [54]	0.50	39.08	6.25	3.87	0.62
Aiello et al. [44]	0.23	38.47	6.20	2.70	0.44
	0.46	50.03	7.07	2.54	0.36
Dorr et al. [56]	0.50	31.30	5.59	1.80	0.32
Average					0.67

^a^ content of fibers added by volume fraction of concrete.

**Table 15:** The ratio between compressive strength and flexure strength for RSF concrete.

Author	Fiber	*fc*′	√*fc′*	FS	FS	FS/√(*fc*′)
	% ^a^	MPa	-	MPa	% ^b^	-
Rossli and Ibrahim [37]	0.20	51.99	7.21	5.45	101.76	0.76
	0.40	59.17	7.69	7.49	139.79	0.97
	0.60	34.50	5.87	6.24	116.58	1.06
	0.80	40.13	6.33	6.36	118.73	1.00
	1.00	28.05	5.30	6.32	118.04	1.19
Siraj and Kedir [47] (Mix-I, C-25)	0.50	27.97	5.29	6.84	111	1.29
	0.50	28.20	5.31	6.96	113	1.31
	0.50	28.23	5.31	8.19	133	1.54
	1.00	27.94	5.29	7.17	116	1.36
	1.00	29.09	5.39	7.89	128	1.46
	1.00	27.72	5.26	10.08	163	1.91
	1.50	28.95	5.38	8.07	131	1.50
	1.50	31.35	5.60	8.64	140	1.54
	1.50	41.03	6.41	13.71	222	2.14
Siraj and Kedir [47] (Mix-II, C-40)	0.50	40.92	6.40	8.34	108	1.30
	0.50	41.51	6.44	8.64	112	1.34
	0.50	41.58	6.45	9.24	120	1.43
	1.00	41.32	6.43	8.46	110	1.32
	1.00	43.66	6.61	9.60	124	1.45
	1.00	41.06	6.41	12.57	163	1.96
	1.50	41.78	6.46	8.70	113	1.35
	1.50	43.94	6.63	9.60	124	1.45
	1.50	58.40	7.64	14.79	192	1.94
Siraj and Kedir [47] (Mix-III, C-60)	0.50	58.98	7.68	8.85	101	1.15
	0.50	60.45	7.77	9.09	104	1.17
	0.50	56.25	7.50	9.21	105	1.23
	1.00	60.56	7.78	9.30	107	1.20
	1.00	60.52	7.78	12.00	137	1.54
	1.00	59.50	7.71	12.84	147	1.66
	1.50	62.41	7.90	11.37	130	1.44
	1.50	59.17	7.69	13.53	155	1.76
	1.50	33.61	5.80	16.14	185	2.78
Abdul Awal et al. [53]	1.00	58.20	7.63	5.75	125	0.75
	1.50	59.00	7.68	6.15	134	0.80
	2.00	42.91	6.55	6.95	151	1.06
Skarżyński and Suchorzewski [54]	0.50	39.08	6.25	4.67	130	0.75
Average						1.39

^a^ content of fibers added by volume fraction of concrete, ^b^ percent increase or decrease in strength of RSF concrete w.r.t plain concrete (100%).

## Data Availability

Not applicable.
